# Supplementary material for: Spontaneous symmetry breaking in polar fluids
Source: Nat Commun. 2024 Jul 11;15:5845. doi: 10.1038/s41467-024-50230-2 (PMC11239904; doi:10.1038/s41467-024-50230-2)
Supplement: Supplementary file 1 — Supplementary Information [file 41467_2024_50230_MOESM1_ESM.pdf]

Spontaneous Symmetry Breaking in Polar Fluids

Supplemental Information

Calum J. Gibb [1], Jordan L. Hobbs [2], Diana I. Nikolova[2], Thomas Raistrick[2], Stuart R. Berrow[2], Alenka Mertelj[3], Natan Osterman[3,4], Nerea Sebastián [3], Helen F. Gleeson[2], and Richard. J. Mandle* [1,2]

[1] School of Chemistry, University of Leeds, Leeds, UK, LS2 9JT
[2] School of Physics and Astronomy, University of Leeds, Leeds, UK, LS2 9JT
[3] Jožef Stefan Institute, Ljubljana, Slovenia
[4] University of Ljubljana, Faculty of Mathematics and Physics, Ljubljana, Slovenia

*r.mandle@leeds.ac.uk

Contents

1. **Supplementary methods**
   1.1. Chemical Synthesis
   1.2. Chemical Characterisation Methods
   1.3. Mesophase Characterisation
   1.4. X-ray Scattering
   1.5. Measurement of Spontaneous Polarization (P_S)
   1.6. Polarised Raman Spectroscopy (PRS)
   1.7. Birefringence Measurements
   1.8. Electronic Structure Calculations
   1.9. MD Simulation Setup and Analysis
   1.10. SHG-M and interferometry
   1.11. Transmission Spectra

2. **Supplementary Discussion**
   2.1. Polarized Optical Microcopy Images
   2.2. DSC Cycles
   2.3. X-ray Scattering
   2.4. Additional Spontaneous polarisation (P_S) Studies
   2.5. Selective Reflection Measurements
   2.6. Polarised Raman Spectroscopy
   2.7. DFT calculations and additional MD simulations
   2.8. Second Harmonic Generation (SHG)
   2.9. Chemical Synthesis
      2.9.1. Synthesis of 5-(4-hydroxyphenyl)-2-(difluoro(3,4,5-trifluorophenoxy)methyl)-1,3-difluorobenzene (*i1*)
      2.9.2. General esterification procedure used in the synthesis of 1-4
      2.9.3. Example NMR Spectra

3. **Supplementary References**

1 Experimental methods

1.1. Chemical Synthesis

Chemicals were purchased from commercial suppliers (Fluorochem, Merck, ChemScene, Ambeed) and used as received. Solvents were purchased from Merck and used without further purification. Reactions were performed in standard laboratory glassware at ambient temperature and atmosphere and were monitored by TLC with an appropriate eluent and visualised with 254 nm light. Chromatographic purification was performed using a Combiflash NextGen 300+ System (Teledyne Isco) with a silica gel stationary phase and a hexane/ethyl acetate gradient as the mobile phase, with detection made in the 200-800 nm range. Chromatographed materials were subjected to re-crystallisation from an appropriate solvent system.

1.2. Chemical Characterisation Methods

The structures of intermediates and final products were determined using ^{1}H, $^{13}C\{^{1}H\}$, and ^{19}F NMR spectroscopy. NMR spectroscopy was performed using either a Bruker Avance III HDNMR spectrometer operating at 400 MHz, 100.5 MHz or 376.4 MHz (^{1}H, $^{13}C\{^{1}H\}$ and ^{19}F, respectively) or a Bruker AV4 NEO 11.75T spectrometer operating at 500 MHz, 125.5 MHz or 470.5 MHz (^{1}H, $^{13}C\{^{1}H\}$ and ^{19}F, respectively).High resolution mass spectrometry data (HRMS) was collected using a Bruker MaXis Impact spectrometer with a positive ESI source (VIP-HESI); the sample was introduced *via* direct infusion as solution in acetonitrile.

1.3. Mesophase Characterisation

Phase transition temperatures and associated enthalpies of transition for compounds 1-4 were determined by differential scanning calorimetry (DSC) using a TA instruments Q2000 heat flux calorimeter with a liquid nitrogen cooling system for temperature control. Samples were measured with 10 ℃ min^{-1} heating and cooling rates. The transition temperatures and enthalpy values reported are averages obtained for duplicate runs. Phase transition temperatures were measured on cooling cycles for consistency between monotropic and enantiotropic phase transitions, while crystal melts were obtained on heating. Phase identification by polarised optical microscopy (POM) was performed using a Leica DM 2700 P polarised optical microscope equipped with a Linkam TMS 92 heating stage. Samples were studied sandwiched between two untreated glass coverslips.

1.4. X-ray Scattering

X-ray scattering measurements, both small angle (SAXS) and wide angle (WAXS) were recorded using an Anton Paar SAXSpoint 5.0 beamline machine. This was equipped with a primux 100 Cu X-ray source with a 2D EIGER2 R detector. The X-rays had a wavelength of 0.154 nm. Samples were filled into either thin-walled quartz capillaries or held between Kapton tape. Temperature was controlled using an Anton Paar heated sampler with a range of -10 °C to 120 °C and the samples held in a chamber with an atmospheric pressure of <1 mbar. Samples were held at 120 °C to allow for temperature equilibration across the sample and then slowly cooled while stopping to record the scattering patterns. Compounds 2, 3 and 4 were measured using a beam-stop while 1 was measured beam-stopless to allow for the whole pattern to be observed. Background scattering off the sample holders was subtracted from the obtained patterns after being appropriately scaled.

The samples were not formally aligned and so these measurements can be considered as "powder" samples. It should be noted that some spontaneous alignment of the LCs both within the capillaries and between the Kapton tape did occur leading to the classic "lobe" pattern seen in the 2D patterns. 1D patterns were obtained by radially integrating the 2D SAXS patterns. Peak position and FWHM was recorded and then converted into d spacing following Bragg's law. In the tilted smectic phase, the tilt was obtained from:

$$\frac{d_c}{d_A} = cos\,\theta \qquad (1)$$

where d_c is the layer spacing in the tilted smectic phase, d_A is the extrapolated spacing from the non-tilted preceding smectic phase, extrapolated to account for the weak temperature dependence of the preceding phases due to shifts in conformation and order, and θ the structural tilt angle.

1.5. Measurement of Spontaneous Polarization (P_S)

Spontaneous polarisation measurements are undertaken using the current reversal technique [1,2].Triangular waveform AC voltages are applied to the sample cells with an Agilent 33220A signal generator (Keysight Technologies), and the resulting current outflow is passed through a current-to-voltage amplifier and recorded on a RIGOL DHO4204 high-resolution oscilloscope (Telonic Instruments Ltd, UK). Heating and cooling of the samples during these measurements is achieved with an Instec HCS402 hot stage controlled to 10 mK stability by an Instec mK1000 temperature controller. The LC samples are held in 4µm thick cells with no alignment layer, supplied by Instec. The measurements consist of cooling the sample at a rate of 1 Kmin^{-1} and applying a set voltage at a frequency of 20 Hz to the sample every 1 K. The voltage was set such that it would saturate the measured P_S and was determined before final data collection.

There are three contributions to the measured current trace: accumulation of charge in the cell (I_c), ion flow (I_i), and the current flow due to polarisation reversal (I_p). To obtain a P_S value, we extract the latter, which manifests as one or multiple peaks in the current flow, and integrate as:

$$P_S = \int \frac{I_p}{2A} dt \qquad (2)$$

where A is the active electrode area of the sample cell. For the N, N_X and, to a lesser extent, the N_F phase, significant amounts of ion flow is present. For materials and mixtures that showed a paraelectric N phase followed by the anti-ferroelectric N_X phase, the N phase always showed some pre-transitional polarisation as well as the significant ion flow mentioned previously. Since the following phase was anti-ferroelectric, this pre-transitional polarisation was anti-ferroelectric in character and so was decoupled from the ion flow in the same way as the N_X phase and as such the P_S of the N and N_X phases was obtained by integrating the peak least affected by ion flow and then doubled to get the total area under both peaks [3].

For the various polar smectic phases found in these materials generally we observed low charge accumulation and ion flow allowing for the baseline to be easily defined and the integrated area of the peak or peaks to be obtained accurately. However, much like for the N phase preceding the N_X phase, the paraelectric SmA phases showed significant pre-transitional polarisation which was measured in the same way as the various polar smectic

phases. The polarisation character of the pre-transitional polarisation was always the same as the polar phase that followed.

1.6. Polarised Raman Spectroscopy (PRS)

Order parameters were determined via polarized Raman spectroscopy (PRS) as described elsewhere [4,5]. Measurements were performed on 10µm Instec cells with anti-parallel planar surface alignment. Raman spectrometry was performed using a Renishaw invia Raman spectrometer equipped with a 20 mW 532 nm laser and an optical microscope with a 10x objective. Measurements were performed in well-aligned regions of the sample devoid of defects using an exposure time of 3x 30s. The Raman spectra are recorded at angles of the nematic director from 0 ° to 180 ° with respect to the incident laser polarization for parallel and perpendicular polarized backscattered light. The 1606cm^{-1} peak was determined to correspond to the C-C breathing mode of the non-fluorinated phenyl ring of **1** via DFT calculations (detailed in section 1.8 and 2.4). The peak was used to determine order parameters by fitting the intensity of the parallel ($I_{\parallel}$) and perpendicular ($I_{\perp}$) components to the following equations [4]:

$$I_{\parallel}(\theta) \propto \frac{1}{5} + \frac{4r}{14} + \frac{8r^2}{15} + \langle P_2 \rangle \left[\frac{1}{21}(3 + r - 4r^2)(1 + 3\cos(2\theta))\right] + \langle P_4 \rangle \left[\frac{1}{280}(1 - r)^2(9 + 20\cos(2\theta) + 35\cos(4\theta))\right] \qquad (3)$$

$$I_{\perp}(\theta) \propto \frac{1}{15}(1 - r)^2 + \langle P_2 \rangle \left[\frac{1}{21}(1 - r)^2\right] + \langle P_4 \rangle \left[\frac{1}{280}(1 - r)^2(3 - 35\cos(4\theta))\right] \qquad (4)$$

Where r is the differential molecular polarizability ratio, $\langle\ \rangle$ denotes an ensemble average, and P_n is the n_{th} Legendre polynomial function. Generally, fits are performed on the depolarisation ratio, $R(\theta)$, to reduce the dependence of the fitting on the incident laser intensity:

$$R(\theta) = \frac{I_{\perp}(\theta)}{I_{\parallel}(\theta)} \qquad (5)$$

1.7. Birefringence Measurements

Birefringence was measured using a Berek compensator mounted in a Leica DM 2700 P polarised optical microscope. The LC sample of compound **1** was measured in a 10 µm anti-parallel rubbed planar cells purchased from Instec. Alignment quality was good in all the phases preceding the $\mathrm{SmC_P^H}$ phase evidenced by the high level of extension in the positions where the rubbing direction of the filled cell was parallel and perpendicular to the polariser axis. While alignment quality decreased in the $\mathrm{SmC_P^H}$ phase an acceptable dark start was obtained parallel and perpendicular to the polariser axis. With the Berek inserted this change in alignment quality translated to a broadening of the extinction fringes but a measurement of optical retardance, and thus birefringence, was still obtained albeit with slightly larger values of associated error.

Birefringence was converted to optical tilt using the equation [6]:

$$\Delta n_{CHP} = \Delta n_{A(F)}(3cos^2\theta - 1)/2 \qquad (6)$$

where Δn_{CHP} corresponds to the birefringence of the SmC_P^H phase, $\Delta n_{A(F)}$ the extrapolated birefringence of the SmA_F phase and θ the optical tilt.

1.8. Electronic Structure Calculations

Electronic structure calculations were performed using Gaussian G16 revision C.01 [7]. For each input structure we first generated a set of unique low energy conformers using the ETKDGv3 rules-based method [8]; each conformer then underwent geometry optimisation at the B3LYP-GD3BJ/aug-cc-pVTZ level of DFT followed by a frequency calculation to confirm the geometry to be at a minimum. For each molecule we then obtain properties (dipole tensor, polarisability tensor) as the probability weighted average. Use of a single minimum energy conformer typically overestimates the molecular dipole moment (e.g. 11.9 D for **1**) and anisotropic polarisability (55.8 Å^3 for **1**)

1.9. MD Simulation Setup and Analysis

Fully atomistic molecular dynamics (MD) simulations were performed in Gromacs 2019, with parameters modelled using the General Amber Force Field (GAFF). [9] Atomic charges were determined using the RESP method [10] for geometry optimised at the B3LYP/6-31G(d) level of DFT [11,12] using the Gaussian G16 revision c01 software package [45]. Topologies were generated using AmberTools 16 [13,14] and converted into Gromacs readable format with Acpype. [15]
We initially constructed a low-density lattice of 800 molecules of **1** - **4** with random positional and orientational order. Following energy minimization by the steepest decent method we performed short (5 ns) equilibration simulations in the NVE and NVT (T = 600 K) ensembles. We then performed a short 'compression' simulation (25 ns) at 600K with an isotropic barostat (P = 100 Bar) to compress the simulation to a liquid like density (~ 1.1 g cm^3). We then obtained a *polar* nematic configuration by applying a static electric field (1 V nm^{-1}) along the x-axis of the isotropic starting configuration for a total of 50 ns at 600 K and 1 Bar; this configuration was used as a starting point for subsequent simulations. Production MD simulations were performed without the biasing field and employed an anisotropic barostat (pressure of 1 Bar), at temperatures of 400 K (and for **1**, also in 10 K increments up to a maximum of 550 K) for a further 250 ns, unless otherwise noted.
Simulations employed periodic boundary conditions in xyz. Bonds lengths were constrained to their equilibrium values with the LINCS algorithm [16]. During production MD simulations the system pressure was maintained at 1 Bar using an isotropic Parrinello-Rahamn barostat. [17,18]. Compressabilities in xyz dimensions were set to 4.5e-5, with the off-diagonal compressibilities were set to zero to ensure the simulation box remained rectangular. Simulation temperature was controlled with a Nosé–Hoover thermostat. [19,20] Long-range electrostatic interactions were calculated using the Particle Mesh Ewald method with a cut-off value of 1.2 nm. A van der Waals cut-off of 1.2 nm was used. MD trajectories were visualised using PyMOL 4.5. Q-tensor analysis was performed using MDTraj 1.9.8 [21]. Cylindrical distribution functions (CDF) were computed using the *cylindr* code [22]. Simulation densities, dipole moments and volumes were obtained with the *gmx energy* program, with dipole and volume being used to compute spontaneous polarisation.
We calculate the second-rank orientational order parameter <*P*$_2$> *via* the Q-tensor according to eq. (7);

$$Q_{\alpha\beta} = \frac{1}{N}\sum_{m=1}^{N}\frac{3a_{m\alpha}a_{m\beta}-\delta_{\alpha\beta}}{2} \qquad (7)$$

where N is the number of molecules, m is the molecule number within a given simulation, α and β represent the Cartesian x, y and z axes, delta is the Kronecker delta, a is a vector that describes the molecular long axis, which is computed for each monomer as the eigenvector associated with the smallest eigenvalue of the inertia tensor. The director at each frame was defined as the eigenvector associated with the largest eigenvalue of the ordering tensor. The order parameter $\langle P_2 \rangle$ corresponds to the largest eigenvalue of $Q_{\alpha\beta}$, and the biaxial order parameter $\langle B \rangle$ corresponds to the difference between the two smallest eigenvalues. The polar order parameter, $\langle P_1 \rangle$, was calculated as the total dipole moment of the simulation box over the sum of the individual molecular dipoles. The polarization, *P*, was calculated from the total dipole of the box over the volume. For smectic phases formed in MD simulations the layer spacing was determined by first fitting a plane to a set of reference atoms for each molecule (the oxygen atom of the CF_2O group); the fit with the highest R^2 indicates the number of layers per simulation (4 in all cases). The layer spacing was then taken as the average distance between planes in the final 200 ns of the simulation.

1.10. SHG-M and interferometry

SHG investigations were performed using EHC D-type 10 µm thick parallel rubbed cells. SHG microscopy and interferometry imaging is performed using a custom-built sample-scanning microscope. A detailed description of the setup can be found in reference [23]. The laser source is an Erbium-doped fiber laser (C-Fiber A 780, MenloSystems) generating 785 nm, 95 fs pulses at a 100 MHz repetition rate. The average power was adjusted using an ND filter to 30 mW on the sample. A combination of galvo mirrors and a long-working distance objective (Nikon CFI T Plan SLWD, NA 0.3) is used to scan the focused beam in the sample plane. The scanning frequencies are much higher (a few 100 Hz) than the imaging frame rate (a few Hz). A long-working distance 20× objective (Nikon CFI T Plan SLWD, NA 0.3) collects the light coming from the sample. A set of 700 nm short-pass and 400 nm band-pass filters eliminates the fundamental IR light and any possible fluorescence signal. The images are finally acquired using a high-performance CMOS camera (Grasshopper 3, Teledyne Flir) with a typical integration time of 250 ms, dimensions 1920 × 1200 pixels, 12-bit depth, and 0.285 µm/pixel.
A motorized half-waveplate for 800 nm rotates the polarization of the fundamental IR beam in the plane and, jointly with the analyzer in front of the camera, enables to perform polarization-resolved SHG. In the case of SHG-M images, the analyzer was removed to account for all contributions. The setup allows for the insertion of a BBO reference crystal before the sample followed by a Michelson interferometer for time compensation between the reference and the fundamental pulse. The phase of the reference pulse is adjusted finely with a glass plate mounted on a motorized rotator to perform interferometric measurements. SHG-I interferograms are obtained by computing the mean intensity and the standard error of the mean intensity of the areas of interest.
Temperature dependence measurements were performed at 0.5 ^{o}C/min cooling rates by imaging a small area of the sample. Intensity is then computed as the mean intensity of a subregion, which is selected to avoid textural defects. The polarization of the incoming beam was selected along the cells rubbing direction after confirmation that maximum SHG signal is obtained in that case.

1.11. Transmission Spectra

A microscope equipped with a 50x objective (~5µm area of observation) fiber coupled to an Avantes AvaSpec-2048 XL spectrometer which used to record transmission spectra of

samples confined in antiparallel rubbed cells with planar alignment (Instec cell, cell gap of 10um). The sample was cooled into the SmC_P^H phase and an area chosen that had the least defects and no spacer beads. Spectra were then recorded at the indicated temperatures on heating back into SmA_F phase.

2 Additional data and supplemental results and discussion

2.1. Polarized Optical Microcopy Images

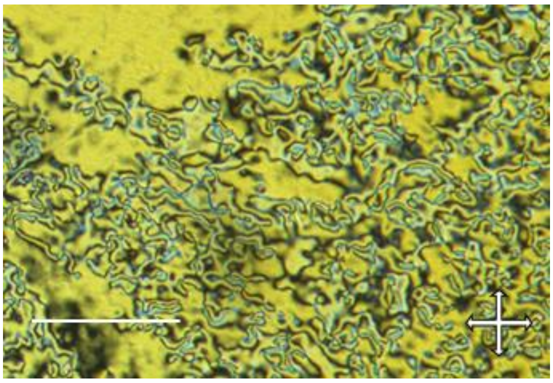

Fig. S1: N phase of compound **1** between untreated coverslips. Scale bar indicates 25 μm

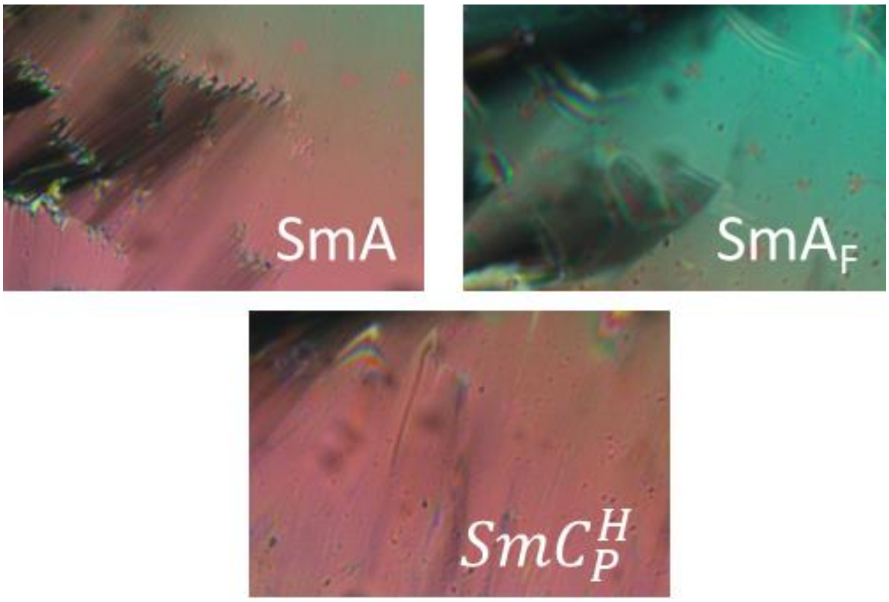

Fig. S2: POM images of **1** in the SmA, SmA$_F$ and SmC_P^H phases showing the same region after cooling. The defects formed between neighbouring fans in the SmA form into regions where the optical retardence rapidly changes showing various birefringence colours. Also clear from these images is how the striated backs of the fans in the SmA texture smooth into uniform areas in the SmA$_F$ phase. These striations return in the SmC_P^H phase and the regions of rapid colour change break into fragmented broken features.

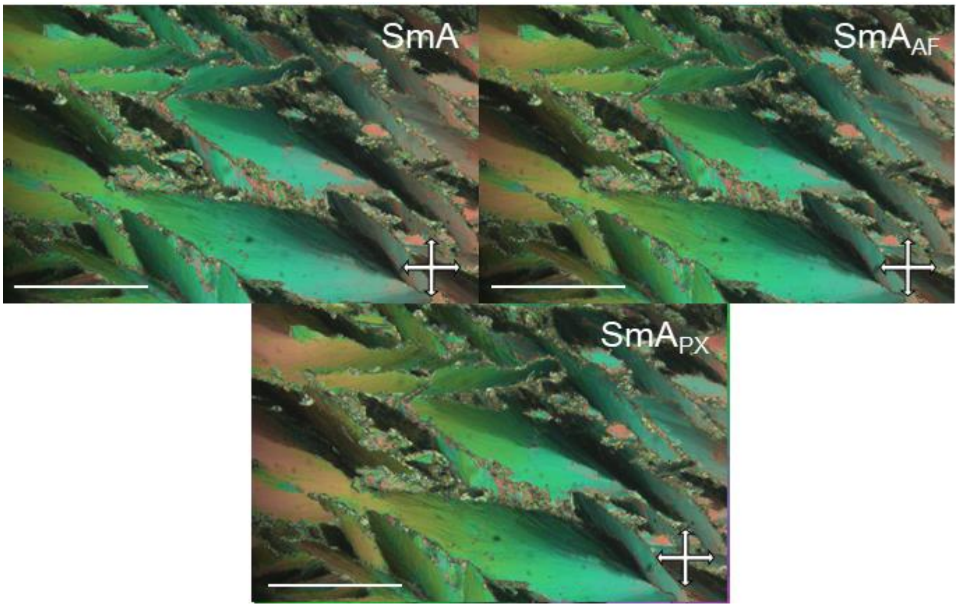

Fig. S3: POM images of **2** in the SmA, SmA$_{AF}$ and SmA$_{PX}$ phases between untreated coverslips. Textural changes between the phases are extremely minimal possibly due to strong peramorphosis. Scale bar indicates 50 µm.

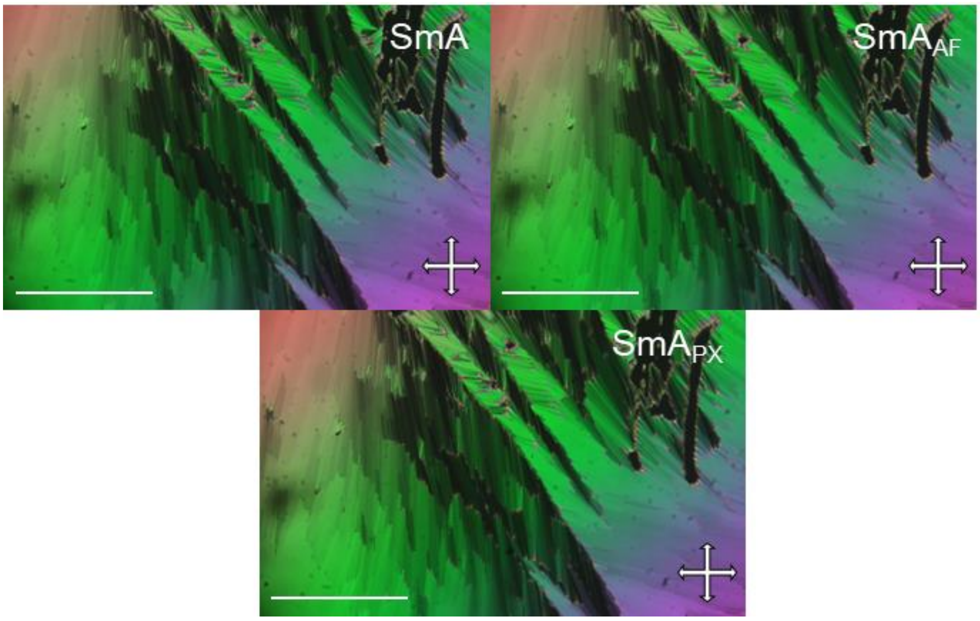

Fig. S4: POM images of **3** in the SmA, SmA$_{AF}$ and SmA$_{PX}$ phases between untreated coverslips. Just as for **2**, the changes in texture between phases are practically non-existent. Scale bar indicates 50 µm.

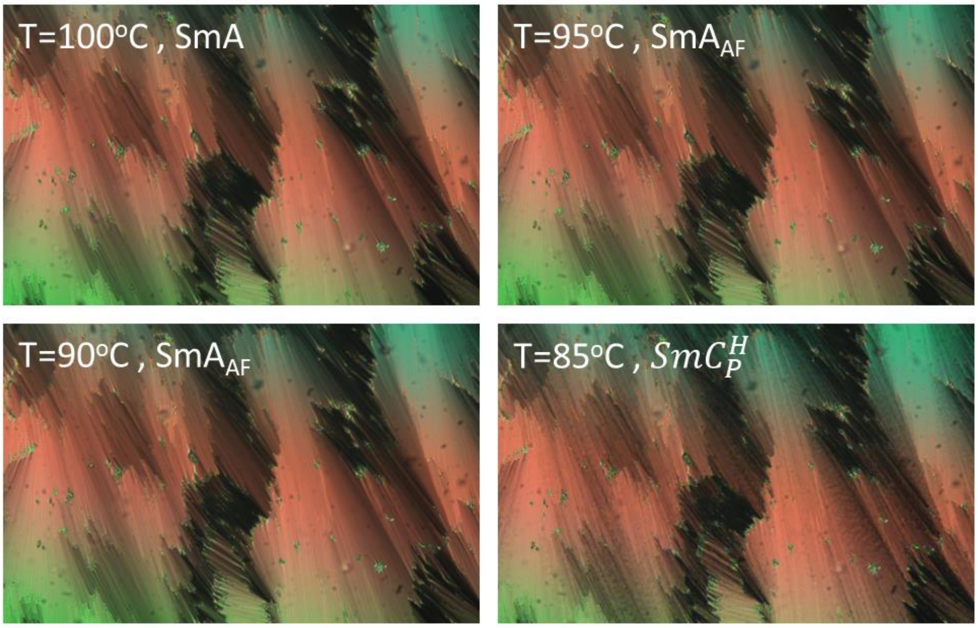

Fig. S5: POM images of **4** in the four smectic phase it exhibits. These images were taken between untreated coverslips on cooling at the indicated temperatures.

2.2. DSC Cycles

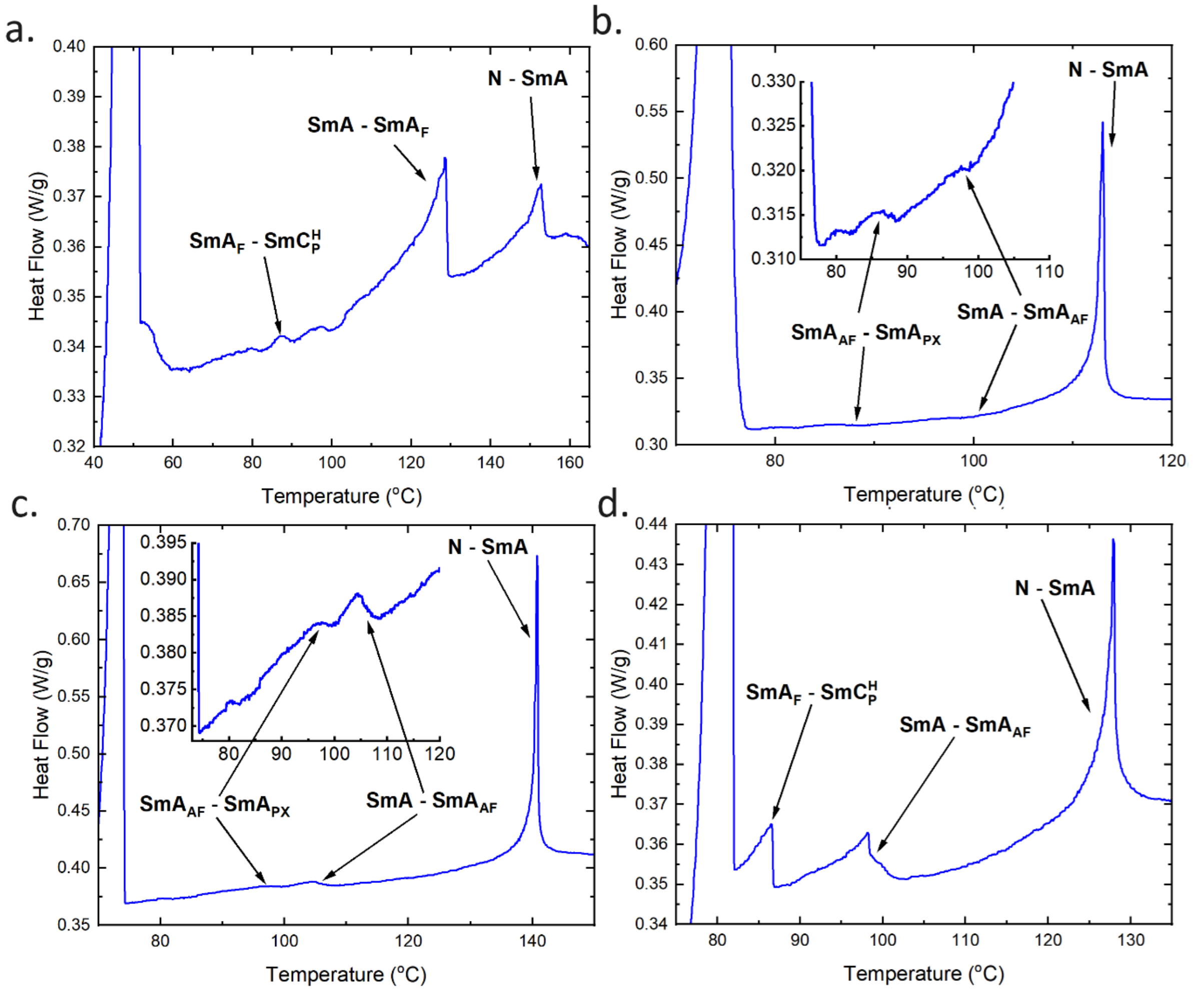

Fig. S6: DSC cycles for the first cooling scan after heating into the nematic phase. (a) **1**, (b) **2**, (c) **3**, and (d) **4**. For also cycles exothermic heat flow is in the upwards direction.

2.3. X-ray Scattering

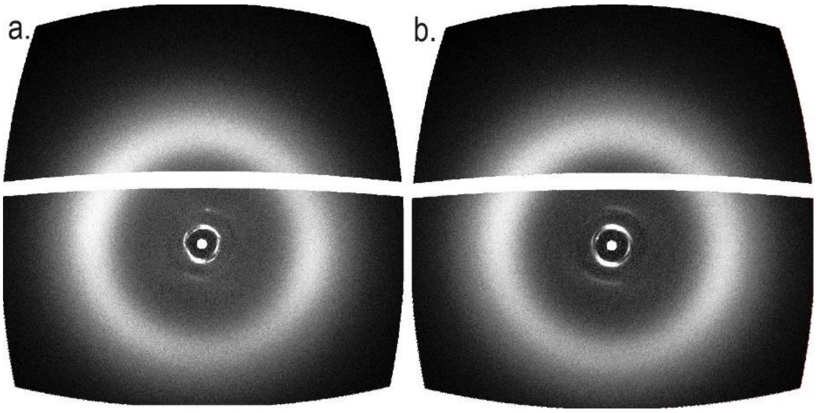

Fig. S7: X-ray scattering patterns of **1** in the (a) SmA_F and (b) SmC_P^H phases.

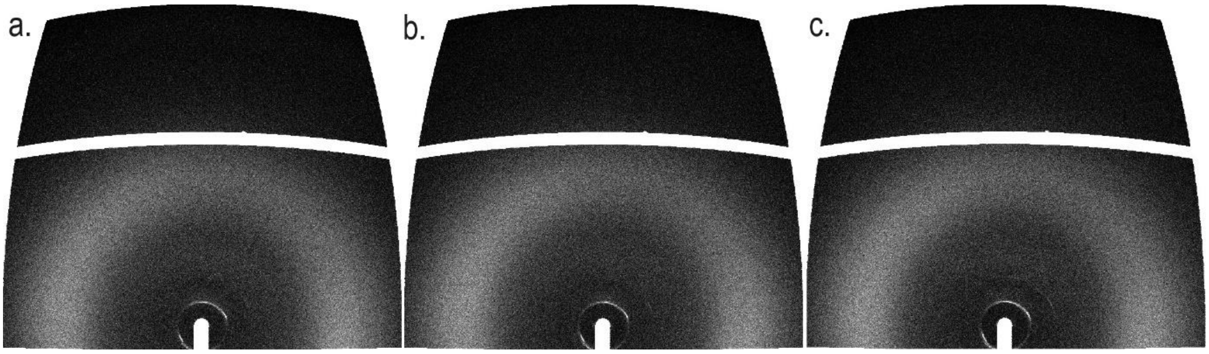

Fig. S8: X-ray scattering patterns of **2** in the (a) SmA, (b) SmA_{AF} and (c) SmA_{PX} phases.

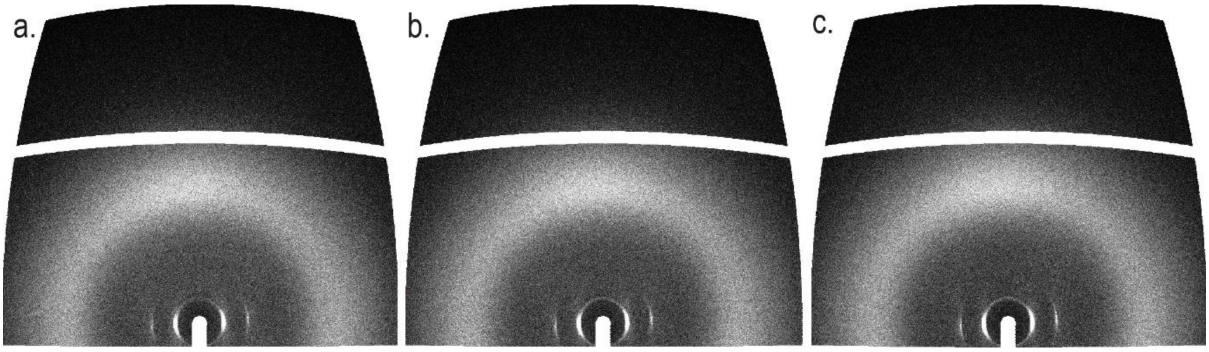

Fig. S9: X-ray scattering patterns of **3** in the (a) SmA, (b) SmA_{AF} and (c) SmA_{PX} phases.

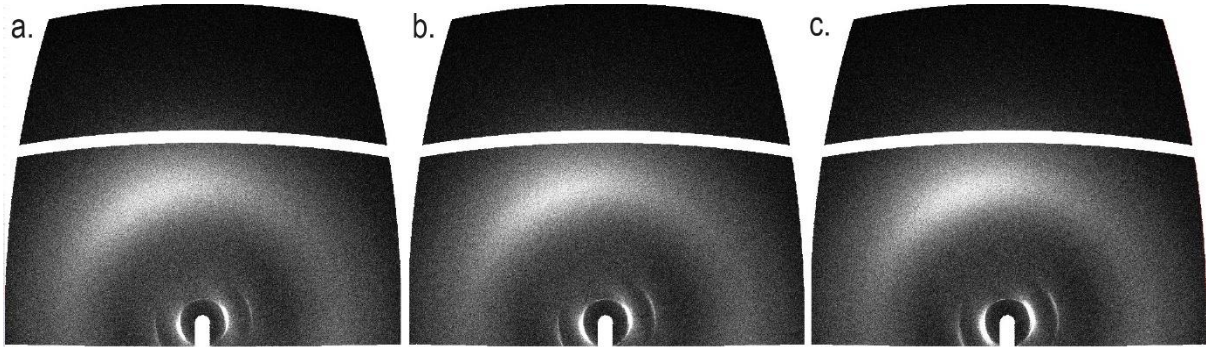

Fig. S10: Two dimensional X-ray scattering patterns of **4** in the (a) SmA, (b) SmA_{AF} and (c) SmC_P^H phases.

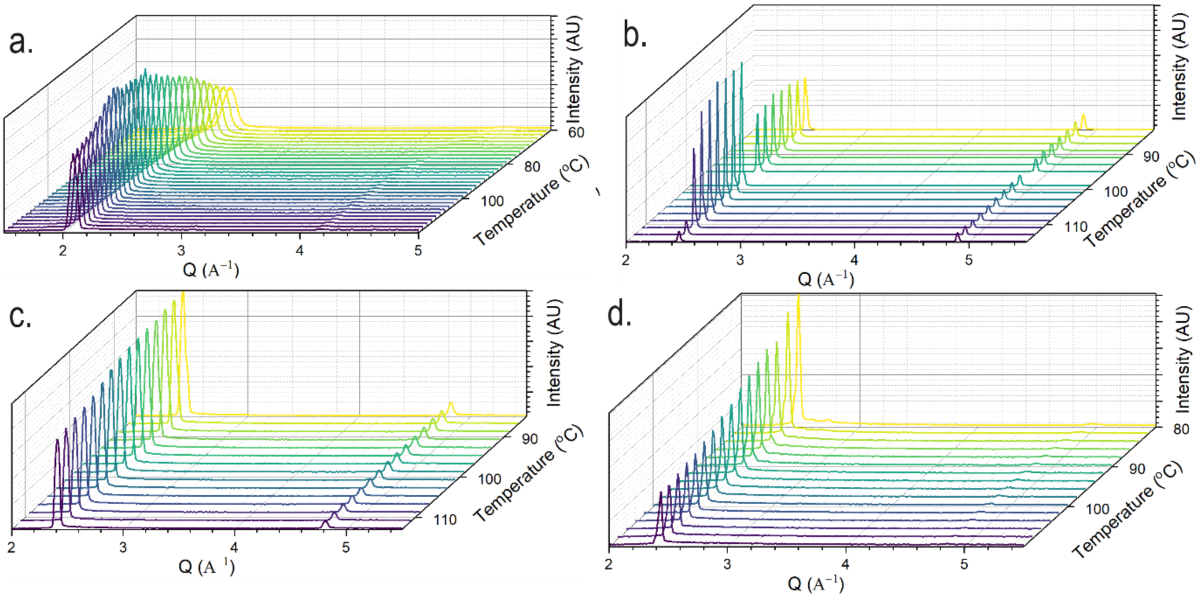

Fig. S11: 1D scattering patterns of (a) **1**, (b) **2**, (c), **3** and (d) **4** as a function of temperature.

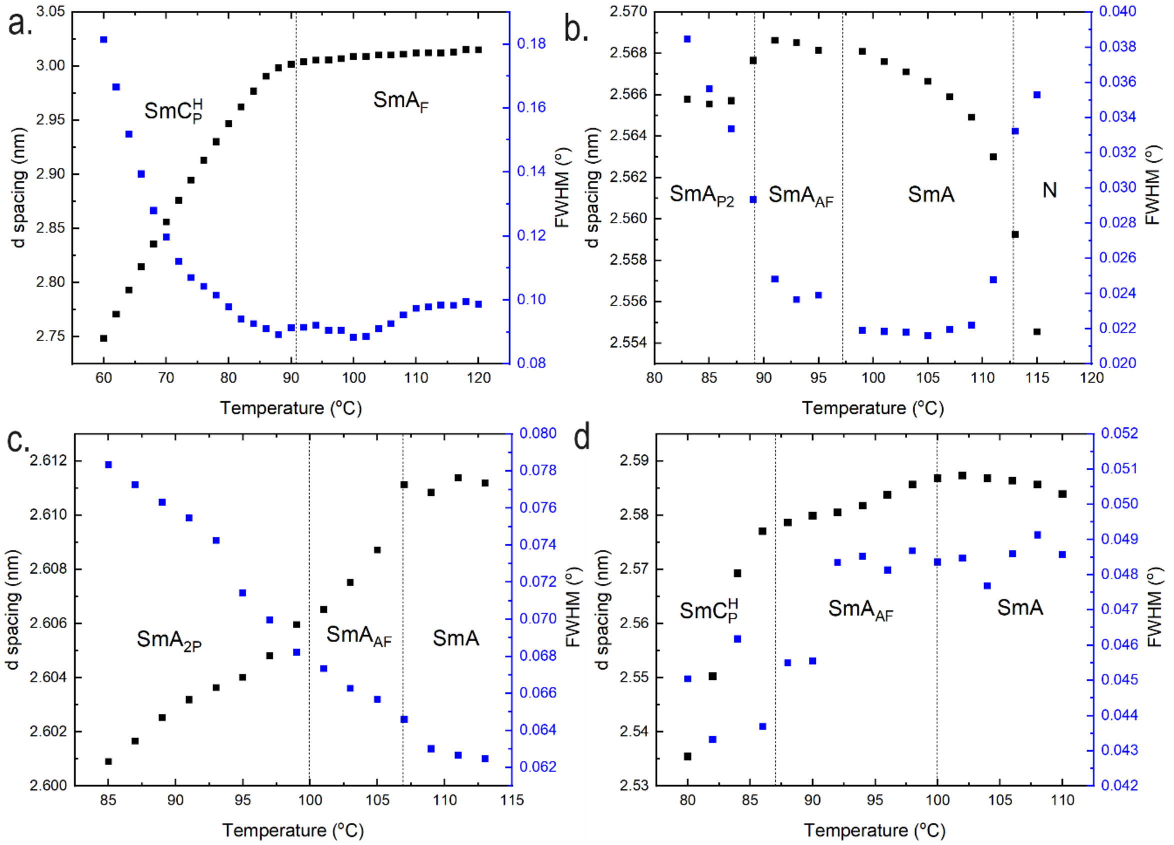

Fig. S12: *d* spacing and FWHM of the small angle peak from Fig. S7 (above) of (a) **1**, (b) **2**, (c), **3** and (d) **4**.

The layer spacing for all the materials in phases we have designated as SmA type are comparable to the average molecular lengths obtained from DFT, indicating monolayer phase types. In the tilted phases (seen in materials **1** and **4**) the d spacing decreases from the DFT value indicating a tilted phase. All the materials show 2nd order Bragg scattering peaks for the small angle peak suggesting significant *pseudo* long range order in the layer spacing, consistent with what has been observed with the ferroelectric nematic phase [24].

2.4. Additional Spontaneous polarisation (P_S) Studies

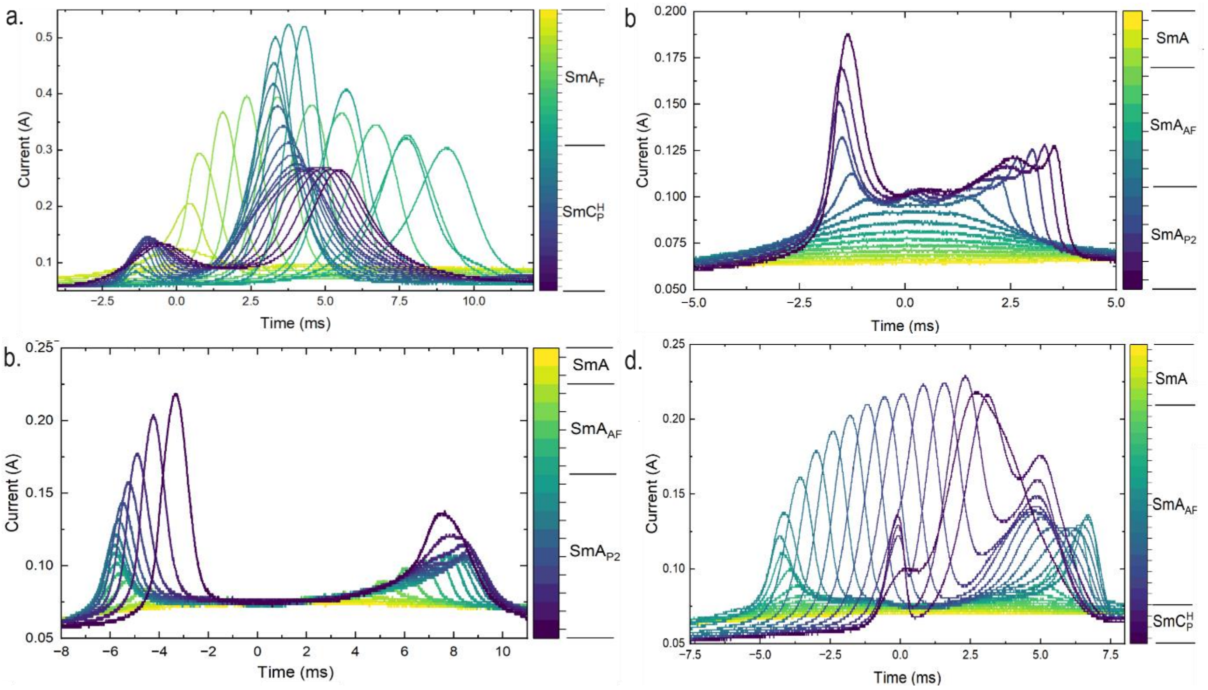

Fig. S13: Current response of the spontaneous polarisation (P_s) for (a) **1**, (b) **2**, (c) **3**, and (d) **4**. All measurements were performed at 20 Hz with the samples confined within a 4-micron cell with no alignment layer.

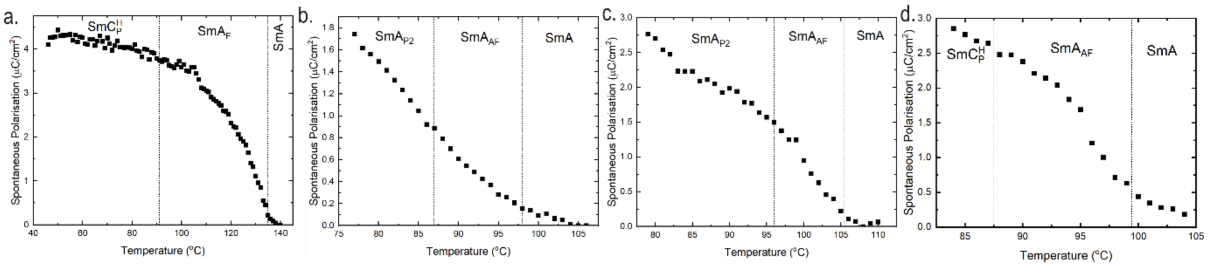

Fig. S14: Measured spontaneous polarisation (P_s) for (a) **1**, (b) **2**, (c) **3**, and (d) **4**. All measurements were performed at 20 Hz with the samples confined within a 4-micron cell with no alignment layer.

The four compounds studied here are show multiple polar smectic phases. Compound **1**, and to a lesser extent **4**, have measured P_S's that are approaching saturation and as such have measured P_S that gives a comparable result to the one obtained from MD simulations (section S2.7, Table S2). The P_S values for compounds **2** and **3** are not saturated at the point of crystallisation and so are not comparable. The SmA_F phase was identified by the observation of only a single peak in the current response. Through the SmA_F phase the time position of the peak continuously moved to longer timescales possibly due to increasing rotational viscosity and coercive voltage [25]. The SmA_{AF} phase was identified by two peaks that are close to symmetric, with one occurring before the applied voltage polarity switch and the other after. The double peaks of the SmA_{AF} phase also showed similar temperature dependence to the time position with the peak at negative times moving to increasingly negative times and the peak in the positive times moving to larger positive, i.e. increasing their separation.

The SmC_P^H phase seemingly has a characteristic P_S trace of a larger peak at longer times with a smaller peak that grows in at times even before the voltage has switched polarity (negative times). This peak is narrower than the longer timescale peak and continuously grows in area, while the area of the larger peak stagnates. However, the total measured polarisation grows following the same temperature dependence unaffected by the shift in relative area of the peaks. The peaks also shift in their time position both moving to more positive timescale with the smaller slower peak being able to shift to the positive times. The smaller peak appears to be associated with the tilt, fig. S15a and S15b demonstrate the strong correlation between the tilt angle (from X-ray data) and the Ps associated with the small peak. Conversely, the Ps contribution of the larger peak stagnates at the phase transition from the SmA_F phase (~90˚C) and does not increase beyond a value of ~3.5mC cm^{-2}. Although there are many possible contributions to Ps curves known for ferroelectric liquid crystals constrained in devices (see [26,27]), this strong correlation leads us to speculate that the initial peak is due to the tilt reforming as the applied voltage is reduced.

Fig. S15c demonstrates the switching mechanism. In the initial part of the curve, where the field is high strength and negative polarity, the system is effectively in the SmA_F configuration. As the field is reduced to lower strength but still negative polarity, the tilt reforms giving back the SmC_P^H structure. Upon increasing the field to across the switching threshold in the positive polarity domain the polarisation of the material fully switches to match the field resulting in the SmA_F structure again. Such a field-induced reorganisation is necessarily identical to field-induced helical unwinding in this system and is reminiscent of the electrooptical behaviour of the N_{TB} phase [28]. It is possible that the introduction of a helical structure is the cause of the "escape from polar order". Further increasing the polarisation at zero field may cost too much free-energy and so a significant change in the phase structure (i.e. formation of a helix) is cheaper and thus preferable to increasing the longitudinal Ps.

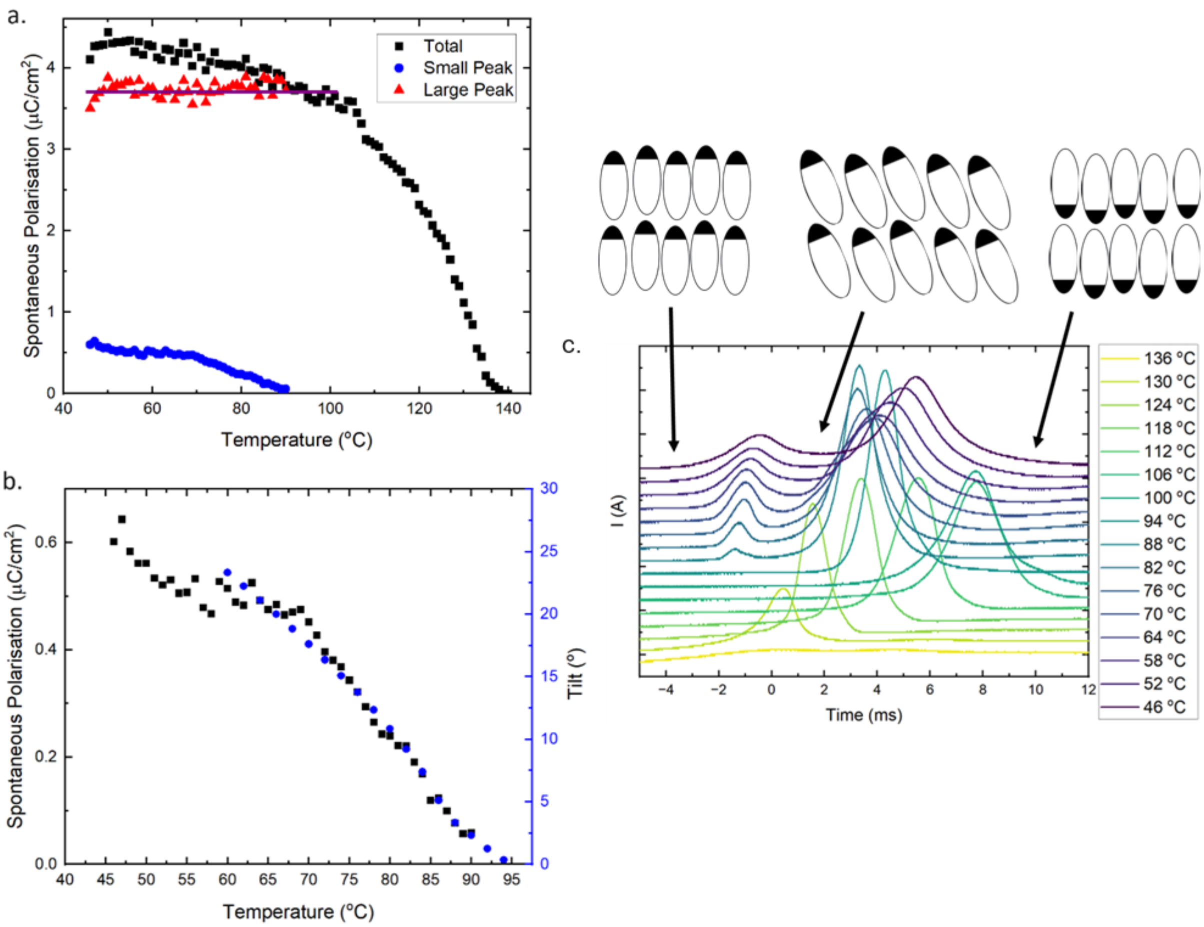

Fig. S15: (a) Measured polarisation for **1**. "Large" and "small" peaks indicate the larger and smaller peak in the current response for material **1** in the $\mathrm{SmC}_\mathrm{P}^\mathrm{H}$ phase. The burgundy line is a guide for the eye and not the result from any fitting. (b) Measured polarisation of the small peak and tilt obtained via X-ray scattering showing the strong correlation between the data. (c) Mechanism for polarisation switching for this material as described in the text. The dark tips of the "molecules" indicates the direction of polarisation. The transition from the SmA_F phase to the $\mathrm{SmC}_\mathrm{P}^\mathrm{H}$ phase occurs at ~90 °C.

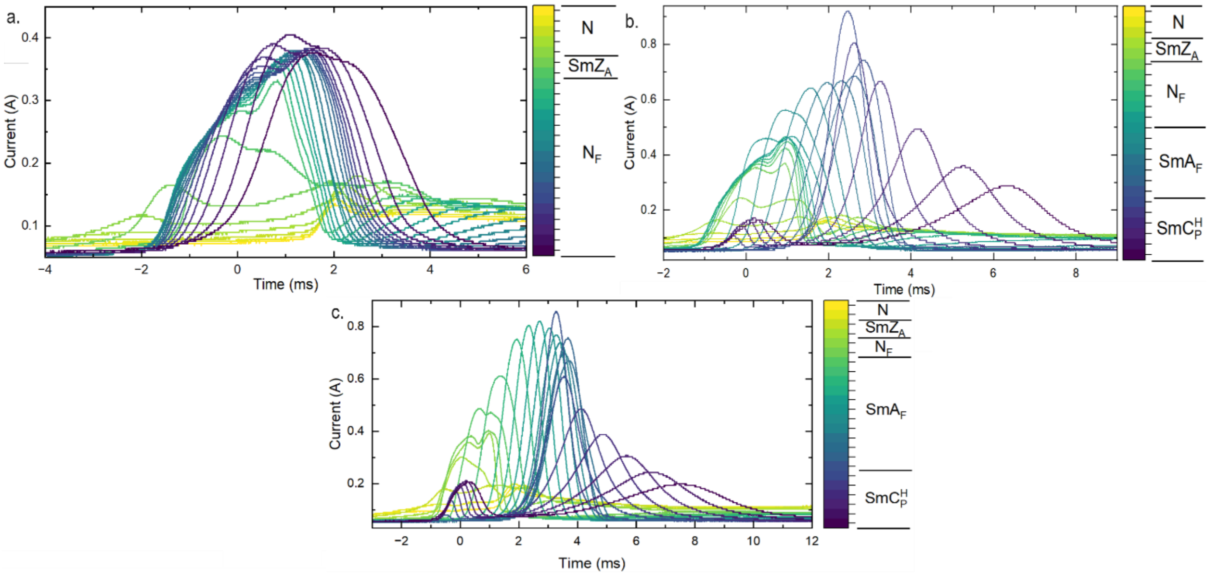

Fig. S16: Current response traces of **1** and DIO in molar ratios of: (a) 40:60, (b) 50:50 and (c) 60:40. All measurements were performed at 20 Hz with a voltage that saturated the measured P_s and the samples confined within a 4-micron cell with no alignment layer.

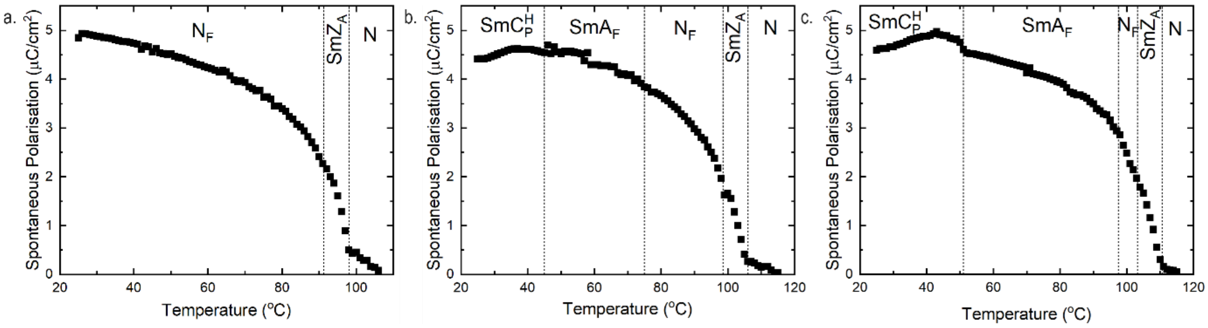

Fig. S17: Temperature dependence of spontaneous polarisation (P_s) of **1** and DIO in molar ratios of: (a) 40:60, (b) 50:50 and (c) 60:40. All measurements were performed at 20 Hz with the samples confined within a 4-micron cell with no alignment layer.

2.5. Selective Reflection Measurements

Measuring the selective reflection of **1** using a spectrometer proved extremely difficult. Selective reflection is observed when the incoming light is parallel to the direction of the periodicity and as such to measure selective reflection homeotropic alignment is required which was found to be unobtainable using polyimide SE1211 (AWAT) or bare ITO electrodes (Instec). However, while good planar alignment was obtained for the N, SmA and SmA_F phase, the SmC_P^H is not uniformly aligned (fig. S22) and so part of the incoming light can get selectively reflected before it is then scattered on the defects and irregularities of the sample, which is seen as the defects appearing bluish. This scattered light is the one detected in our setup. The results in fig. S18 indicate that the pitch length decreases with decreasing temperature before saturating at some amount. #

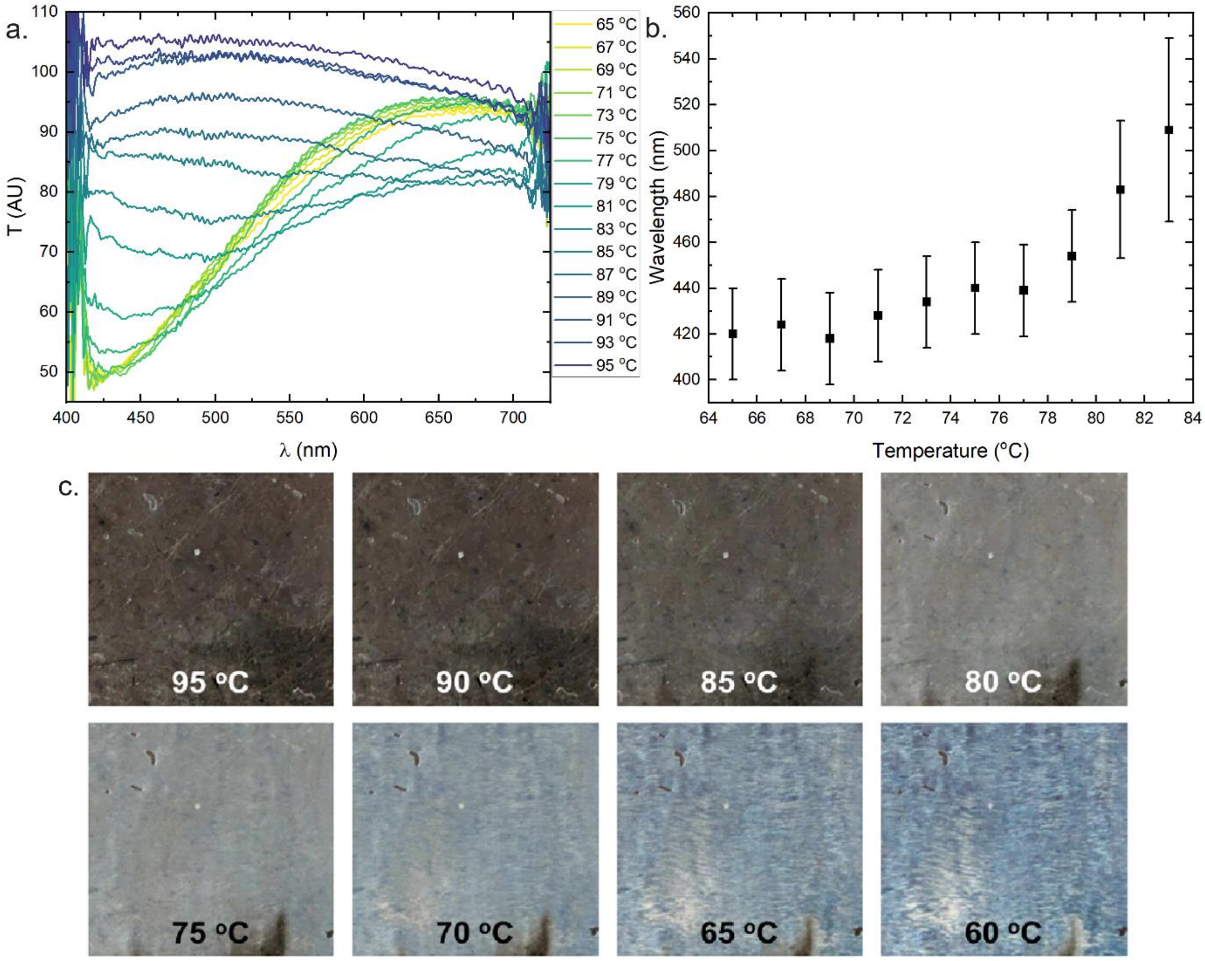

Fig. S18: (a) transmission spectra of **1** in a parallel planar aligned cell. (b) Peak position from transmission measurements. (c) unpolarised images of the electrode area of the cell usage in the spectroscopic measurements. Above 83 °C the wavelength of the reflection is not clear and is generally seen as an increase in scattering which translates to decreased transmission.

The selective reflection of light was not only observed in pure samples of 1 (Fig. S18a) but also in binary mixtures of **1** and DIO at ambient temperatures. As with the pure material, mixtures comprised of 60% DIO gave large domains of opposite handedness when viewed with circularly polarised light of opposite handedness (Fig. S19b). We were also able to obtain

an image a thin cell with no anchoring condition containing a mixture comprised of 50% DIO showing almost the whole visible range at room temperature (Fig. S19c).

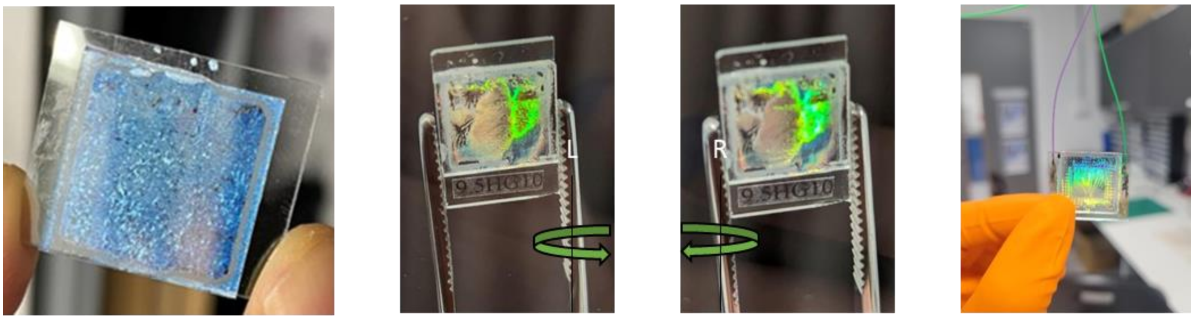

a. b. c.

Fig. S19: (a) Image of **1** confined in a 5-micron cell with no alignment layer showing the selective reflection of light and the many domains of the sample, (b) Images of binary mixtures of 60% 1 and DIO filled into a planar aligned cell taken with a circular polariser of left and right polarisation demonstrating the handedness of the light reflected from the sample. The lack of uniformity across the cell is indicative of various domains of different helical rotation and pitch orientation, and (c) a mixture of 50% **1** and DIO confined within a 10-micron cell with no alignment layer showing the selective reflection of light at room temperature.

2.6. Polarised Raman Spectroscopy

To gain greater insight into the ordering of **1** in the $\mathrm{SmC_P^H}$ phase, Raman spectroscopy was performed to determine the 2nd and 4th rank uniaxial order parameters $\langle P_2 \rangle$ and $\langle P_4 \rangle$. Suggestions of heliconical tilt and/or tilt in general can be observed via a reduction in the determined order parameters $\langle P_2 \rangle_{\mathrm{app}}$ and $\langle P_4 \rangle_{\mathrm{app}}$ upon cooling into the tilted phase [5,29]. The cause of the reduction in $\langle P_2 \rangle_{\mathrm{app}}$ and $\langle P_4 \rangle_{\mathrm{app}}$ needs to be considered within the context of the observed phase. For example, for heliconical phases, it is expected that a reduction in $\langle P_2 \rangle_{\mathrm{app}}$ and $\langle P_4 \rangle_{\mathrm{app}}$ and no change in the director angle, θ_D, as determined via fitting the depolarization ratio data [5,30] provided that the heliconical axis is in the same plane and direction as the original nematic director. It should be noted that for the $\mathrm{N_{TB}}$ phase, close to the N to $\mathrm{N_{TB}}$ transition, no reduction in $\langle P_2 \rangle$ was reported, most likely due to the very small tilt angle in the $\mathrm{N_{TB}}$ phase, minimizing the effect, coupled with a likely small increase in the actual order parameter upon entering the lower temperature phase. However, a reduction in $\langle P_4 \rangle$ was observed and is related to the interplay between tilt and increasing orientational order on cooling [4].

For tilted phases, it is expected to see no reduction in $\langle P_2 \rangle_{\mathrm{app}} = \langle P_2 \rangle$ and $\langle P_4 \rangle_{\mathrm{app}} = \langle P_4 \rangle$ and no change in θ provided that the tilt happens within the plane of the laser polarization vector [31]. If, however, a component of the tilt occurs normal to the plane of the polarization vector, it is expected that a reduction of $\langle P_2 \rangle_{\mathrm{app}}$ and $\langle P_4 \rangle_{\mathrm{app}}$ would be observed. It should be noted that, in general, measurement of $\langle P_2 \rangle$ and $\langle P_4 \rangle$ can be determined in the tilted smectic phases, SmC* and de Vries SmA, but this requires special considerations such as determination of parameters like in plane tilt angle, a modification of the general Raman intensity functions, eq. (3) and eq. (4), for uniaxial singly peaked ODF *ab initio*, or consideration of the alignment to suppress helical structure [32–34]. Fig. S20a shows the director angle as determined by the fitting of depolarization ratio data, it can be seen that the

angle of the director remains effectively constant in any given domain. The tilt in the $\mathrm{SmC}_{\mathrm{P}}^{\mathrm{H}}$ phase measured from SAXS layer spacing measurements, accounts for the reduction in the measured $\langle P_2 \rangle_{\mathrm{app}}$ and $\langle P_4 \rangle_{\mathrm{app}}$ as determined via PRS. As the director angle remains unchanged, it can be concluded that the phase structure is most likely a tilted director that is averaged around a symmetry axis e.g. in a helical manner within the phase.

Once $\langle P_2 \rangle_{\mathrm{app}}$ and $\langle P_4 \rangle_{\mathrm{app}}$ are determined, the heliconical tilt angle, Ψ, can be calculated via the following formula (Fig. S20b) [33,34]:

$$\langle P_n \rangle_{\mathrm{app}} = \langle P_n \rangle P_n \cos(\Psi) \quad (8)$$

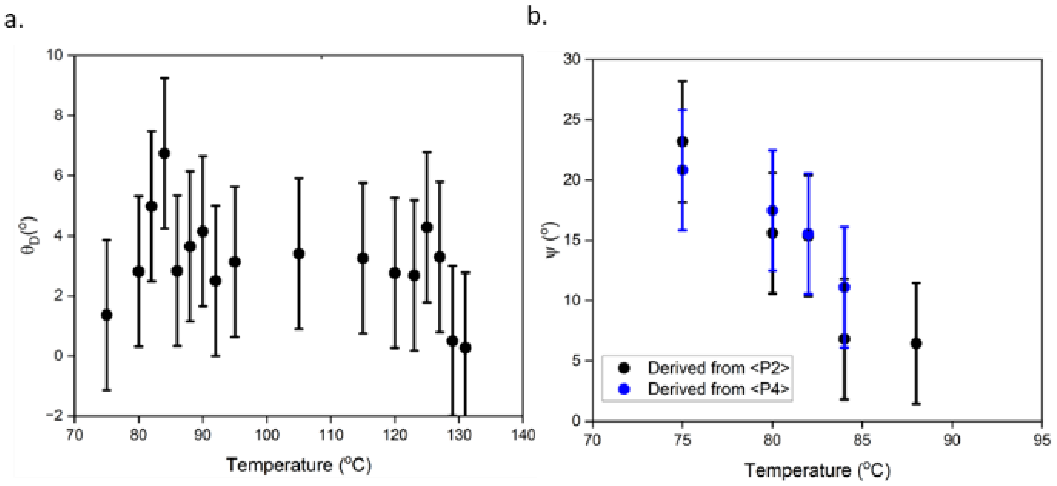

Fig. S20: (a) Director angle as determined via the fitting of the depolarization ratio eq. (5), and (b) Heliconical angle as determined via PRS by fitting to eq. (8).

2.7. DFT calculations and additional MD simulations

For **1**, the large molecular electric dipole moment (11.2 D) implies that the maximum polarisation possible would be ~ 5.5 µC cm^2 (assuming perfect polar order, $\langle P_1 \rangle = 1$, and a mass density of 1.3 g cm^3 [35] which is reasonably close to experimental values (Table S1). For **1** we also computed the Raman spectrum finding it to be in good agreement with the experimental spectrum albeit slightly shifted to higher frequencies. Analysis of the displacement vectors confirms that the 1606 cm^{-1} peak (used in the calculation of $\langle P_2 \rangle$, $\langle P_4 \rangle$) is associated with the breathing mode of the unfluorinated 1,4-disubstituted benzene (and thus is suitable for determination of $\langle P_2 \rangle$ and $\langle P_4 \rangle$, while the peak at 1630 cm^{-1}coresponds to the breathing mode of the adjacent 3,5-difluorobenzene.

Cmp.	No. Conf.	Dipole / D	Dipole Ang / °	α / Å^3	Δα / Å^3
**1**	46	11.2	9.0	62.6	50.8
**2**	33	9.5	12.0	54.5	46.9
**3**	37	9.2	10.0	55.0	48.9
**4**	29	8.5	13.0	54.7	46.4

Table S1: Tabulated properties of compounds **1** - **4** obtained from DFT calculations at the B3LYP-GD3BJ/aug-cc-pVTZ level of DFT given as the probability weighted average over the indicated number of conformers (No. Conf.). The dipole moment is the magnitude of the dipole tensor; Dipole Ang. Is the angle between the dipole moment and the molecular long axis, α and Δα are the isotropic and anisotropic polarizabilities, respectively.

Let us now discuss additional MD simulations of **1** as described in the manuscript. Each simulation commences from a polar nematic starting configuration, however compound **1** readily adopts a lamellar structure and so we observe a polar SmC phase at temperatures up to 430 K, while at 440 K and 450 K we observe a polar SmA phase (Fig. S21c). The transition temperatures are notably overestimated compared to experimental values, yet the ability to reproduce phase type is encouraging. Gratifyingly, the values of polarisation for these simulations are consistent with experimentally obtained values (and are within the limits estimated from DFT calculations), while the value of $\langle P_1 \rangle$ is close to saturated. The values of $\langle P_2 \rangle$ we obtain within each phase are consistent with the trends obtained experimentally by PRS. Depending on the temperature of the MD simulation, we find 1 to variously exhibit polar SmC, polar SmA, polar N, or isotropic phases. To facilitate comparison with experimental data (where this is available) we have offset the simulation temperature in the plots below so that they are relative to the $\mathrm{SmC_P^H}$ to SmA_F transition (which occurs at 90 °C). It should be noted that our MD simulations overestimate this transition temperatures of **1** by around 50 °C. MD simulations competently predict the layer spacing in the SmA phase, as well as the temperature dependent decrease in the SmC phase (Fig. S21c). The polar order parameter $\langle P_1 \rangle$, remains close to saturated at all temperatures. If the orientational order parameter is computed relative to the layer normal, $\langle P_{2layernormal} \rangle$, then it displays the same temperature dependent decrease as seen when measured *via* PRS. Additionally, we also performed MD simulations of compounds **2** - **4** at a temperature of 400 K (Table S2). Whereas **1** forms a polar SmC configuration spontaneously, we find **2** and **3** form a polar SmA phase (equivalent to the experimentally observed SmA_F phase), whereas **4** remains in the polar nematic (equivalent to N_F) configuration. CDF plots were computed for **1** - **4** (Fig. S21e) at 400 K (variously in the polar SmC, SmA and N configurations). The CDF of **1** differs from **2** - **4**, with the head-to-tail separation being at a larger spacing owing to its increased molecular length (due to the additional 2,5-disubstituted 1,3-dioxane ring).

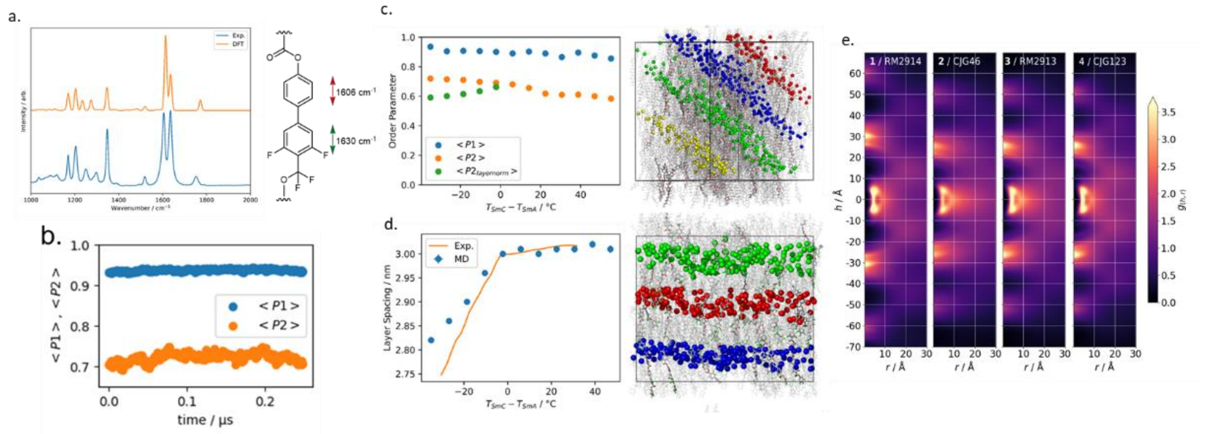

Fig. S21: (a) Plot of the experimental polarised Raman spectrum of **1** in the nematic phase (Exp., blue) and the simulated Raman spectrum of the same compound obtained at the B3LYP-GD3BJ/cc-pVTZ level of DFT (DFT, orange). The simulated spectrum is constructed by adding a Gaussian at each harmonic frequency whose height is the calculated Raman activity and with a FWHM of 15 cm^{-1}. The calculated and experimental spectra are offset for clarity, (b) lot of the polar (<P1>) and orientational (<P2>) order parameters as a function of simulation time, showing that both are sustained in the absence of the biasing field, (c+d) MD simulations for **1** showing plots of layer spacing and order parameters (polar, <P1>; orientational, <P2> and <$P2_{layernormal}$>) as a function of reduced temperature (defined as T_{SmC} – T_{SmA}) for (a) the polar SmC phase and (b) the polar SmA phase. The molecules are shown as wire frame, with the oxygen atom of the –CF_2O- group shown as a sphere to illustrate the layered structure and coloured according to the layer number, and (c) Cylindrical distribution functions (CDF) for 1-4 at 400 K In the polar SmC (1), SmA (2, 3) and N (4) configurations.

Cmp.	Phase	<P1>	<P2>	Ps / C m^2	ρ /kg m^3	d / nm
**1**	SmC	0.936 ± 0.003	0.718 ± 0.01	0.053 ± 0.0	1336.138 ± 2.452	2.8 ± 0.1
**2**	SmA	0.861 ± 0.006	0.647 ± 0.012	0.047 ± 0.0	1311.284 ± 3.612	2.5 ± 0.1
**3**	SmA	0.880 ± 0.005	0.670 ± 0.013	0.046 ± 0.0	1294.389 ± 3.254	2.4 ± 0.1
**4**	N	0.922 ± 0.004	0.692 ± 0.011	0.045 ± 0.0	1271.97 ± 3.387	n/a

Table S2: Tabulated simulation properties for compounds **1** - **4**, where *Phase* is the simulation phase type judged from inspection of the simulation, <P1> is the polar order parameter, <P2> is the orientational order parameter, P_S is the spontaneous polarization, ρ is the density, and d is the layer spacing. All simulations were performed at 400 K and commenced from a polar nematic starting configuration, and in all cases polar order was retained over the entire production MD simulation (250 ns).

2.8. Second Harmonic Generation (SHG)

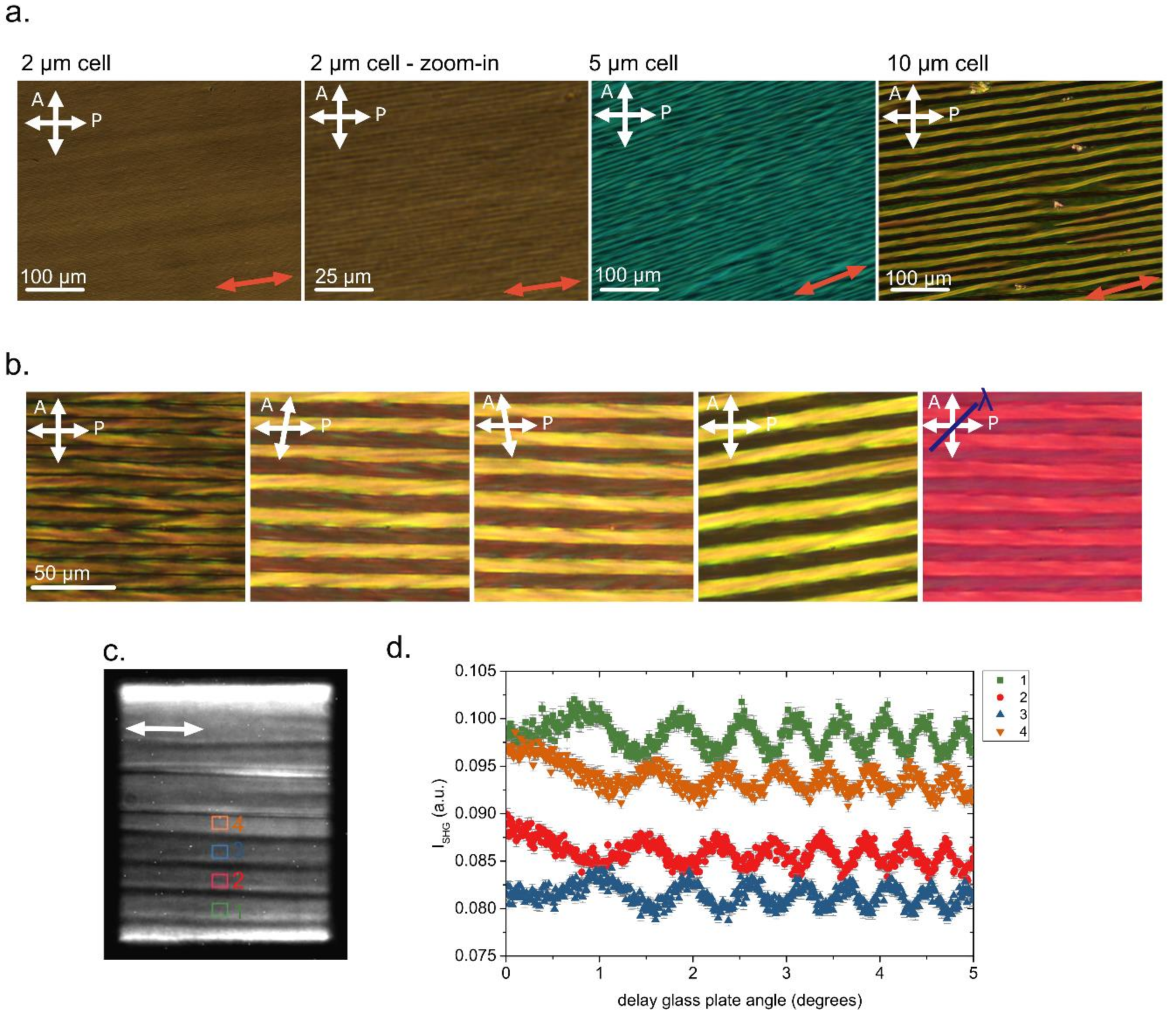

Fig. S22. a) Compound **1** in parallel rubbed cells. Textures observed between crossed polarizers for cells of different thicknesses. Starting from the left, 2 µm, 2 µm zoom-in image, 5 µm and 10 µm. Double headed orange arrow marks the rubbing direction of the cell. b) Pom textures for the 10 µm cell at different conditions: crossed polarizers, analyser uncrossed anticlockwise, analyser uncrossed clockwise, sample rotated 8 degrees anticlockwise and sample in extinction position with lambda plate inserted at 45 degrees. Textures reveal opposite optical activity for two consecutive ribbons with extinction obtained at ± 8 degrees. c) SHG microscopy image of the ribbon-like superstructure in 10 µm cell. d) SHG interferogram for the areas highlighted in (c) corresponding to 4 adjacent ribbons. Interferogram shows alternating phase from one ribbon to the next one, indicating opposite polarization direction. Double headed white arrow indicates direction of polarization of the incoming IR beam.

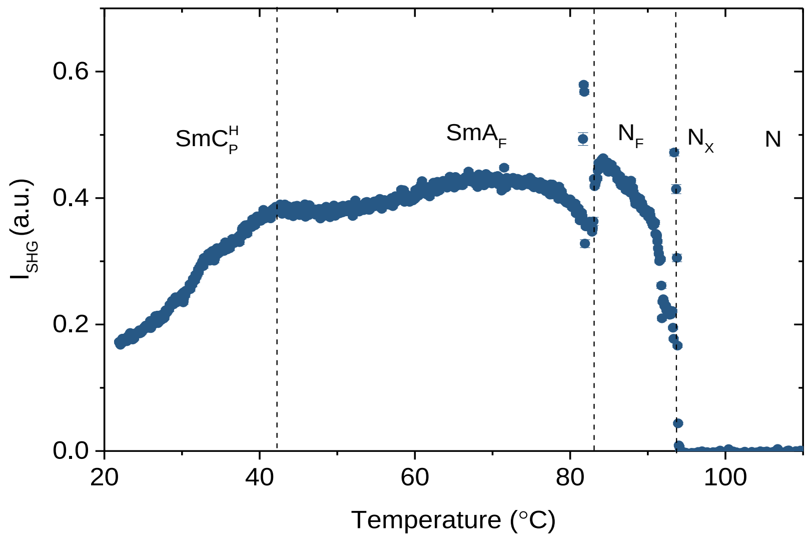

Fig. S23. Temperature dependence of SHG signal for a 45:55 mixture of compound **1** and DIO.

2.9. Chemical Synthesis

Materials 1-4 were synthesised as outlined in Scheme S1 as part of a wider library of materials that exhibit the phase types described herein. A Suzuki-Miyaura cross-coupling of 5-bromo-2-(difluoro(3,4,5-trifluorophenoxy)methyl)-1,3-difluorobenzene with 4-hydroxybenzene boronic acid pinacol ester afforded 5-(4-hydroxyphenyl)-2-(difluoro(3,4,5-trifluorophenoxy)methyl)-1,3-difluorobenzene (*i1*) in high yield. Subsequent esterification with a selection of carboxylic acids afforded compounds **1 – 4**.

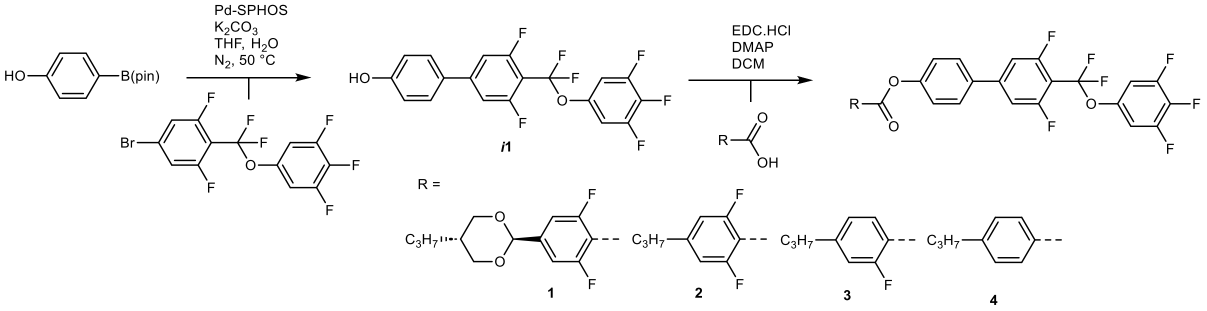

Fig. S24: Synthetic scheme for the preparation of materials 1-4.

2.9.1. Synthesis of 5-(4-hydroxyphenyl)-2-(difluoro(3,4,5-trifluorophenoxy)methyl)-1,3-difluorobenzene (*i1*)

A solution of 5-bromo-2-(difluoro(3,4,5-trifluorophenoxy)methyl)-1,3-difluorobenzene (7.78 g, 20 mmol) in a biphasic mixture of THF (60 mL) and 2M aqueous K_2CO_3 (60 mL, 2M) was degassed by sparging with argon. Separately, 10 mL of THF was degassed by sparging with argon; solid $Pd(OAc)_2$ (1 mmol) and SPHOS (2 mmol) were added, the suspension was stirred for 5 minutes to afford a solution of Pd-SPHOS in THF. The biphasic reaction solution was heated under reflux under an atmosphere of dry argon; 4-hydroxyphenyl boronic acid pinacol ester (4.84 g, 22 mmol) was added in one portion, followed by the solution of Pd-SPHOS in THF. The reaction was heated under reflux for 12 h, at which point TLC showed complete consumption of the starting pinacol ester. The solution was then cooled to ambient temperature, the aqueous layer separated and washed with ethyl acetate (3x 50 ml) and discarded. The combined organics were sequentially washed with saturated aqueous ammonium carbonate (50 ml), brine (50 ml). The organics were then dried over $MgSO_4$, filtered, and volatiles removed in vacuo. The crude material was filtered over a short plug of silica gel, eluting with DCM, and then recrystalised from ethanol, affording the title compound.

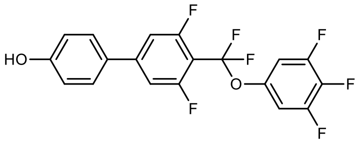

Yield: 7.2 g (89 %, off white solid);
R_f (DCM): 0.33
^{1}H NMR (400 MHz, $CDCl_3$): 5.70 (1H, S, Ar-OH), 6.96 (2H, ddd, $J_{H\text{-}H}$ = 2.4 Hz, $J_{H\text{-}H}$ = 2.8 Hz, $J_{H\text{-}H}$ = 8.4 Hz, Ar-H), 6.97 - 7.02 (2H, m, Ar-H), 7.15-7.21 (2H, m, Ar-H), 7.49 (2H, ddd, $J_{H\text{-}H}$ = 2.4 Hz, $J_{H\text{-}H}$ = 2.8 Hz, $J_{H\text{-}H}$ = 8.4 Hz, Ar-H).
^{19}F NMR (376 MHz, $CDCl_3$): -163.27 (1F, *tt*, $J_{H\text{-}F}$ = 6.1 Hz, $J_{F\text{-}F}$ = 21.0 Hz, Ar-F), -132.54 (2F, *dd,* $J_{H\text{-}F}$ = 8.7 Hz, $J_{F\text{-}F}$ = 20.5 Hz, Ar-F), -110.71 (2F, *td*, $J_{H\text{-}F}$ = 11.5 Hz, $J_{F\text{-}F}$ = 26.2 Hz, Ar-F), -61.51 (2H, *t*, $J_{F\text{-}F}$ = 26.2 Hz, CF_2O).z

2.9.2. General esterification procedure used in the synthesis of 1-4

A round bottomed flask or 14ml vial was charged with 5-(4-hydroxyphenyl)-2-(difluoro(3,4,5-trifluorophenoxy)methyl)-1,3-difluorobenzene (*i1*) (402 mg, 1 mmol), the appropriate carboxylic acid (1.1 mmol), EDC.HCl (1.5 mmol) and DMAP (0.1 mmol). Dichloromethane was added (conc. ~ 0.1 M) and the suspension stirred until complete consumption of the phenol as judged by TLC. The volatiles were removed in vacuo and the crude material was subjected to flash chromatography over silica gel with a gradient of hexane/DCM using a Combiflash NextGen 300+ System (Teledyne Isco). The materials were subsequently recrystallized from EtOH before being dried under reduced pressure to give the reported yields.

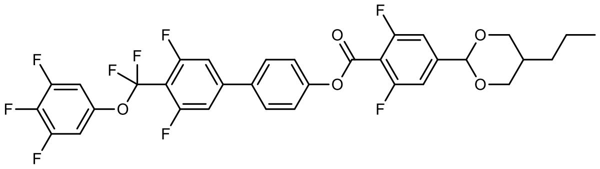

Yield: 490 mg (74 %, colourless crystals);
R_f (DCM): 0.90
^{1}H NMR (500 MHz, $CDCl_3$): 7.62 (ddd, *J* = 8.7, 2.6, 1.9 Hz, 2H, Ar-H), 7.39 (ddd, *J* = 8.6, 2.7, 2.0 Hz, 2H, Ar-H), 7.24 – 7.16 (m, 4H. Ar-H), 7.04 – 6.94 (m, 2H, Ar-H), 5.40 (s, 1H, Ar-CH-$(O)_2$), 4.26 (dd, *J* = 7.0, 4.6 Hz, 2H, O-CH_{axial}(H)-CH), 3.54 (t, *J* = 11.4 Hz, 2H, O-CH_{eqit}(H)-CH), 2.15 (m, 1H, $(CH_2)_2$-CH-CH_2), 1.35 (h, *J* = 7.2 Hz, 2H, CH_2-CH_2-CH_3), 1.11 (q, *J* = 7.2 Hz, 2H, CH-CH_2-CH_2), 0.94 (t, *J* = 7.3 Hz, 3H, CH_2-CH_3).

$^{13}C\{^1H\}$ NMR (125 MHz, $CDCl_3$): 162.17 (d, *J* = 6.4 Hz), 160.51 (dd *J* = 257.1 Hz, *J* = 5.9 Hz), 159.61 (t, *J* = 2.3 Hz), 151.43 (dd *J* = 257.2 Hz, *J* = 5.8 Hz),151.25, 150.94 (dd *J* = 251.5 Hz, *J* = 6.2 Hz), 145.99 (t, *J* = 10.8 Hz), 145.44 (t, *J* = 10.8 Hz), 144.44 – 144.85 (m), 138.49 (dt, *J* = 249.7 Hz, *J* = 13.6 Hz), 135.52 (m), 128.24, 122.50, 111.06 (dd, *J* = 24.1 Hz, *J* = 2.9 Hz), 110.26 (dd, *J* = 23.8 Hz, *J* = 3.3 Hz), 109.49 – 110.09 (m), 107.84 - 107.14 (m), 99.84, 72.59, 33.90, 30.23, 19.53, 14.18.

$^{19}F\{^1H\}$ NMR (470.5 MHz, $CDCl_3$): -61.66 (t, *J* = 26.1 Hz, CF_2O), -108.69 (d, *J* = 9.8 Hz, Ar-F), -110.06 (td, *J* = 26.2, 11.0 Hz, Ar-F), -132.46 (dd, *J* = 20.8, 8.7 Hz, Ar-F), -163.16 (tt, *J* = 21.1, 5.9 Hz, Ar-F).

HRMS: 671.1464 (calcd. for $C_{33}H_{24}F_9O_5$: 671.1475, err +1.5 ppm, M + H)
693.1297 (calcd. for $C_{33}H_{23}F_9NaO_5$: 693.1294, err -0.4 ppm, M + Na)

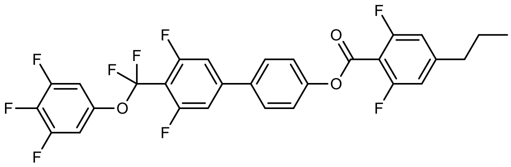

(**2**) Ex 19

Yield: 414 mg (71 %, white crystals);
R_f (DCM): 0.80
^{1}H NMR (500 MHz, $CDCl_3$): 7.62 (d, *J* = 8.8 Hz, 2H, Ar-H), 7.38 (d, *J* = 8.7 Hz, 2H, Ar-H), 7.22 (d, *J* = 10.3 Hz, 2H, Ar-H), 7.04 – 6.94 (m, 2H, Ar-H), 6.87 (d, *J* = 9.3 Hz, 2H, Ar-H), 2.65 (t, *J* = 7.3 Hz, 2H, Ar-CH_2-CH_2), 1.68 (h, *J* = 7.5 Hz, 2H, CH_2-CH_2-CH_3), 0.97 (t, *J* = 7.4 Hz, 3H, CH_2-CH_3).

$^{13}C\{^1H\}$ NMR (125 MHz, $CDCl_3$): 162.18 (d, *J* = 5.6 Hz), 160.31 (dd *J* = 256.4 Hz, *J* = 5.94 Hz), 160.15 (d, *J* = 5.4 Hz), 159.99 (m), 151.31, 151.21 (dd *J* = 256.0 Hz, *J* = 5.4 Hz), 151.12 (dd *J* = 256.1 Hz, *J* = 5.8 Hz), 150.84 (t, *J* = 9.4 Hz), 146.02 (t, *J* = 9.8 Hz), 144.88 – 144.55 (m), 135.39 (m), 128.23, 122.55, 112.25 (dd, *J* = 23.0 Hz, *J* = 3.1 Hz), 111.26-110.92 (m), 107.62 – 107.32 (m), 37.87, 32.66, 13.56

^{19}F NMR (470.5 MHz, $CDCl_3$): -61.66 (t, *J* = 26.2 Hz, CF_2O), -109.64 (d, *J* = 10.1 Hz, Ar-F), -110.07 (td, *J* = 26.3, 11.3 Hz, Ar-F), -132.46 (dd, *J* = 20.6, 8.7 Hz, Ar-F), -163.14 (tt, *J* = 20.7, 6.0 Hz, Ar-F).

HRMS: 585.1107 (calcd. for $C_{29}H_{18}F_9O_3$: 585.1107, err +0.0 ppm, M + H)
607.0922 (calcd. for $C_{29}H_{17}F_9NaO_3$: 607.0926, err +0.7 ppm, M + Na)

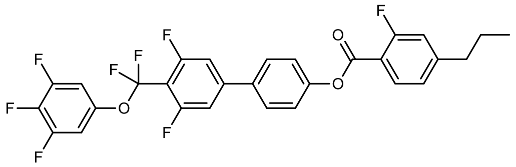

(**3**)

Yield: 340 mg (62 %, colourless crystals);
R_f (DCM): 0.95
^1H NMR (400 MHz, $CDCl_3$) :7.95 (t, *J* = 7.8 Hz, 1H, Ar-H), 7.54 (ddd, *J* = 8.8, 2.7, 2.1 Hz, 2H, Ar-H), 7.28 (ddd, *J* = 8.8, 2.7, 2.2 Hz, 2H, Ar-H), 7.17 – 7.09 (m, 2H, Ar-H), 7.05 – 7.00 (m, 1H, Ar-H), 7.00 – 6.96 (m, 1H, Ar-H), 6.97 – 6.87 (m, 3H, Ar-H), 2.60 (tz, *J* = 8.5, 6.8 Hz, 2H, Ar-CH_2-CH_2), 1.62 (h, *J* = 7.5 Hz, 2H, CH_2-CH_2-CH_3), 0.90 (t, *J* = 7.4 Hz, 3H, CH_2-CH_3).

$^{13}C\{^1H\}$ NMR (101 MHz, $CDCl_3$): 163.79, 162.62 (d, *J* = 5.6 Hz), 161.18, 160.36 (d, *J* = 254.2 Hz, *J* = 5.6 Hz), 152.10 (d, *J* = 9.8 Hz), 151.67, 150.88 (dd *J* = 254.7 Hz, *J* = 5.6 Hz), 150.71 (dd *J* = 254.7 Hz, *J* = 5.8 Hz), 146.11 (t, *J* = 9.4 Hz), 145.74-144.50 (m), 140.04-139.51 (m), 137.42-136.94 (m), 132.41, 128.17, 124.47 (d, *J* = 3.0 Hz), 126.66, 117.04 (d, *J* = 21.8 Hz), 114.92 (d, *J* = 9.3 Hz), 111.02 (dd, *J* = 24.2 Hz, *J* = 2.9 Hz), 107.64 – 107.24(m), 37.85, 23.89, 13.66

^{19}F NMR (376 MHz, $CDCl_3$): -61.64 (t, *J* = 26.3 Hz, CF_2O), -108.49 (dd, *J* = 11.9, 7.6 Hz, Ar-H), -110.13 (td, *J* = 26.3, 11.0 Hz, Ar-H), -132.46 (dd, *J* = 20.6, 8.6 Hz, Ar-H), -163.15 (tt, *J* = 20.7, 5.9 Hz, Ar-H).

HRMS: 567.1203 (calcd. for $C_{29}H_{19}F_8O_3$: 567.1201, err -0.4 ppm, M + H)
589.1021 (calcd. for $C_{29}H_{18}F_9NaO_3$: 589.1020, err -0.1 ppm, M + Na)

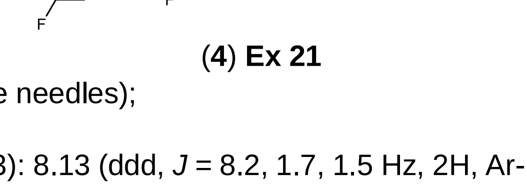

(**4**) **Ex 21**

Yield: 470 mg (86 %, white needles);
R_f (DCM): 0.83
1H NMR (400 MHz, CDCl3): 8.13 (ddd, *J* = 8.2, 1.7, 1.5 Hz, 2H, Ar-H), 7.63 (ddd, *J* = 8.7, 2.9, 1.9 Hz, 2H, Ar-H), 7.39 – 7.30 (m, 4H, Ar-H), 7.22 (m, 2H, Ar-H), 7.05 – 6.95 (m, 2H, Ar-H),

2.70 (t, J = 6.8 Hz, 2H, Ar-CH_2-CH_2),), 1.70 (h, J = 7.2 Hz, 2H, CH_2-CH_2-CH_3), 0.98 (t, J = 7.3 Hz, 3H, CH_2-CH_3).

$^{13}C\{^{1}H\}$ NMR (101 MHz, $CDCl_3$): 165.07, 160.30 (dd, J = 258.0 Hz, J = 5.8 Hz), 152.05, 151.88 (dd J = 257.4 Hz, J = 5.8 Hz), 151.76 (dd J = 257.1 Hz, J = 5.8 Hz), 149.47 (m), 146.18 (t, J = 9.4 Hz), 146.84-146.44 (m), 135.39 (m), 128.23, 122.55, 112.25 (dd, J = 23.0 Hz, J = 3.1 Hz), 130.33, 128.83, 128.17, 126.63, 111.01 (dd, J = 23.9 Hz, J = 2.9 Hz), 107.65 – 107.21 (m), 38.15, 24.26, 13.75

^{19}F NMR (376 MHz, $CDCl_3$): -61.64 (t, J = 26.3 Hz, CF_2O), -110.14 (td, J = 26.2, 11.1 Hz, Ar-H), -132.45 (dd, J = 20.6, 8.7 Hz, Ar-H), -163.15 (tt, J = 21.0, 5.9 Hz, Ar-H).

HRMS: 549.1298 (calcd. for $C_{29}H_{20}F_7O_3$: 549.1295, err -0.4 ppm, M + H)
571.1109 (calcd. for $C_{29}H_{19}F_7NaO_3$: 571.1115, err 0.9 ppm, M + Na)

2.9.3. Example NMR Spectra

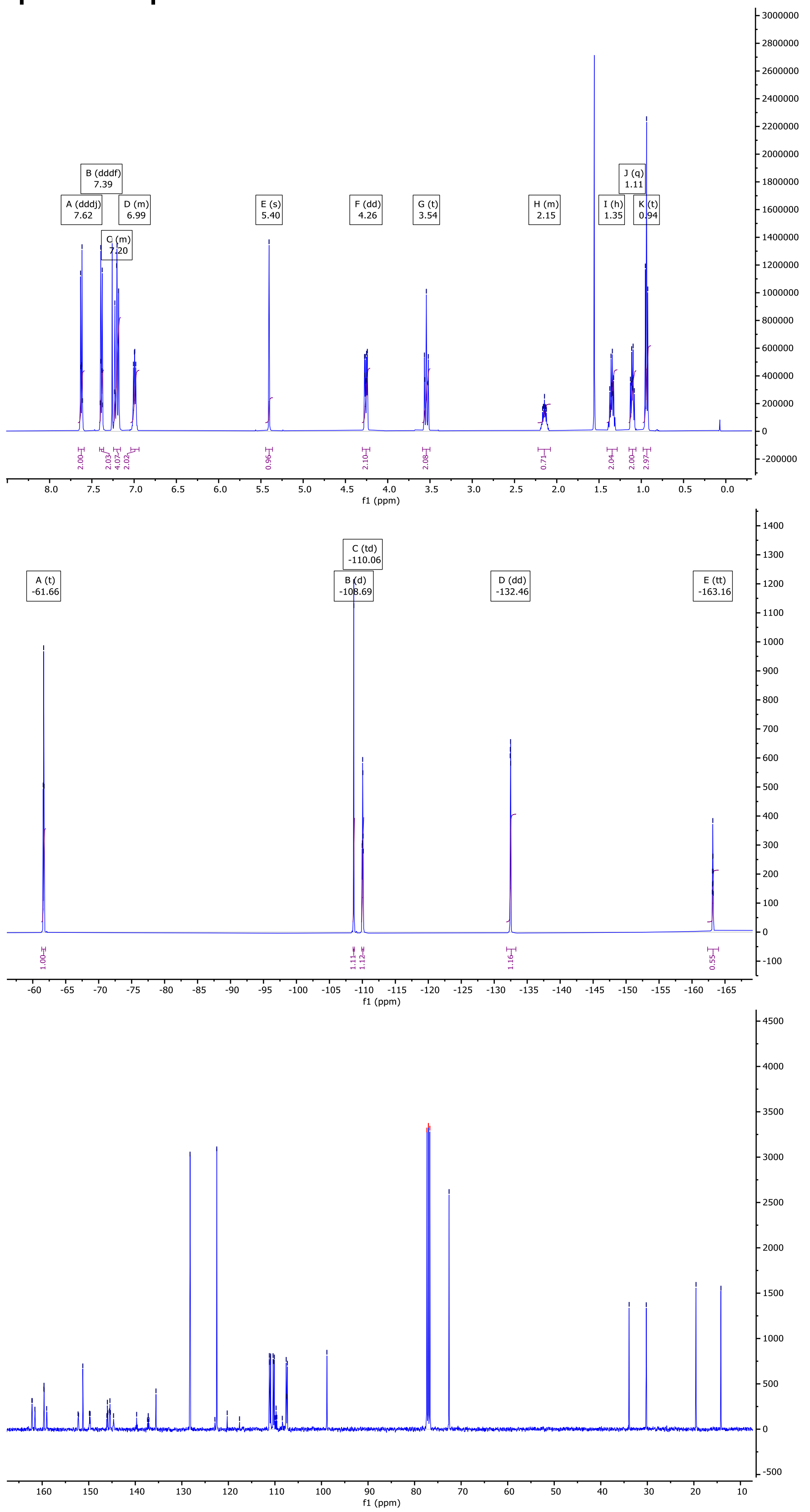

Fig. S25: Example NMR spectra of material **1**: ^{1}H [top], ^{19}F [middle], and ^{13}C{^{1}H} [bottom].

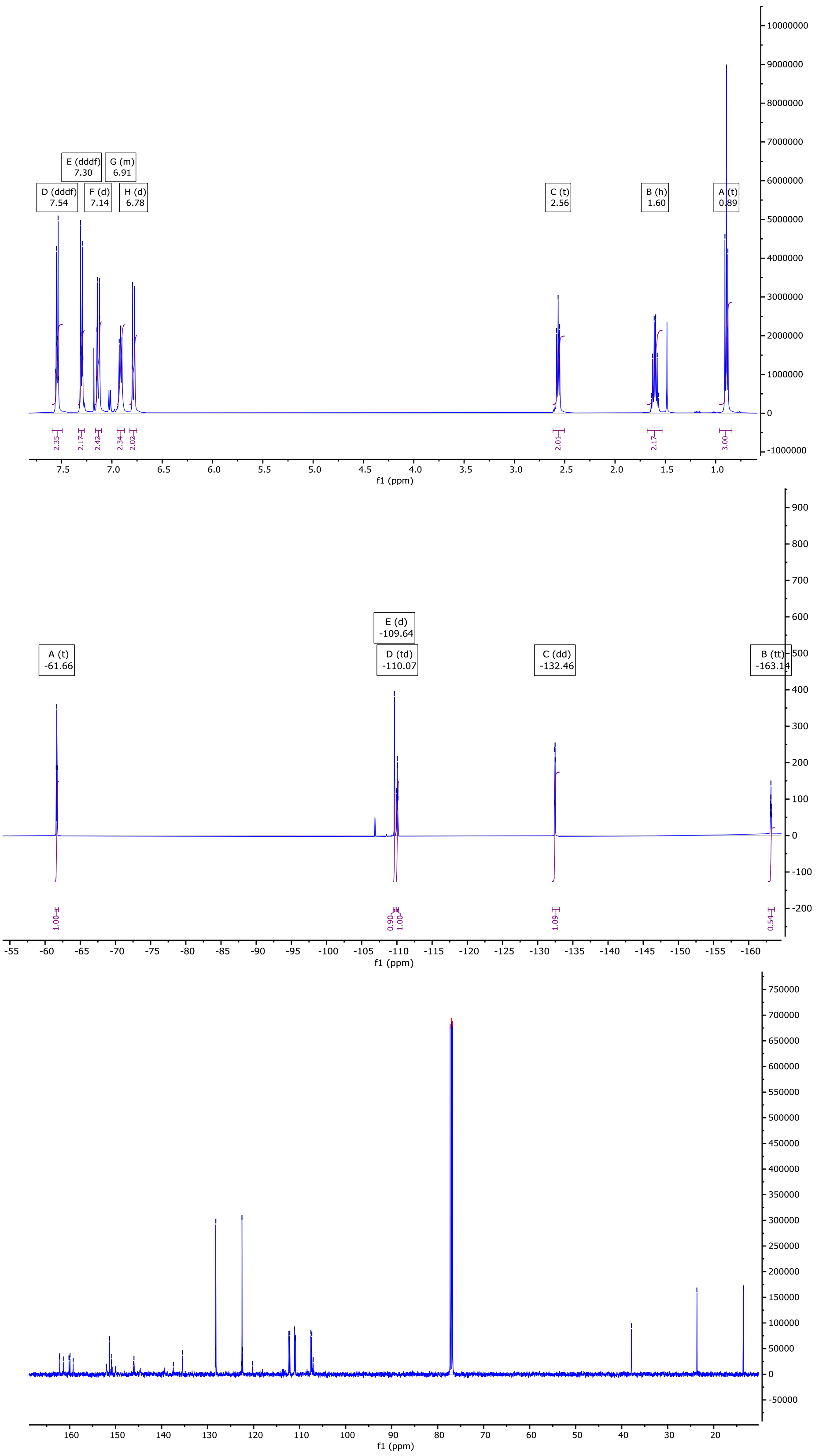

Fig. S26: Example NMR spectra of material **2**: ^{1}H [top], ^{19}F [middle], and $^{13}C\{^{1}H\}$ [bottom].

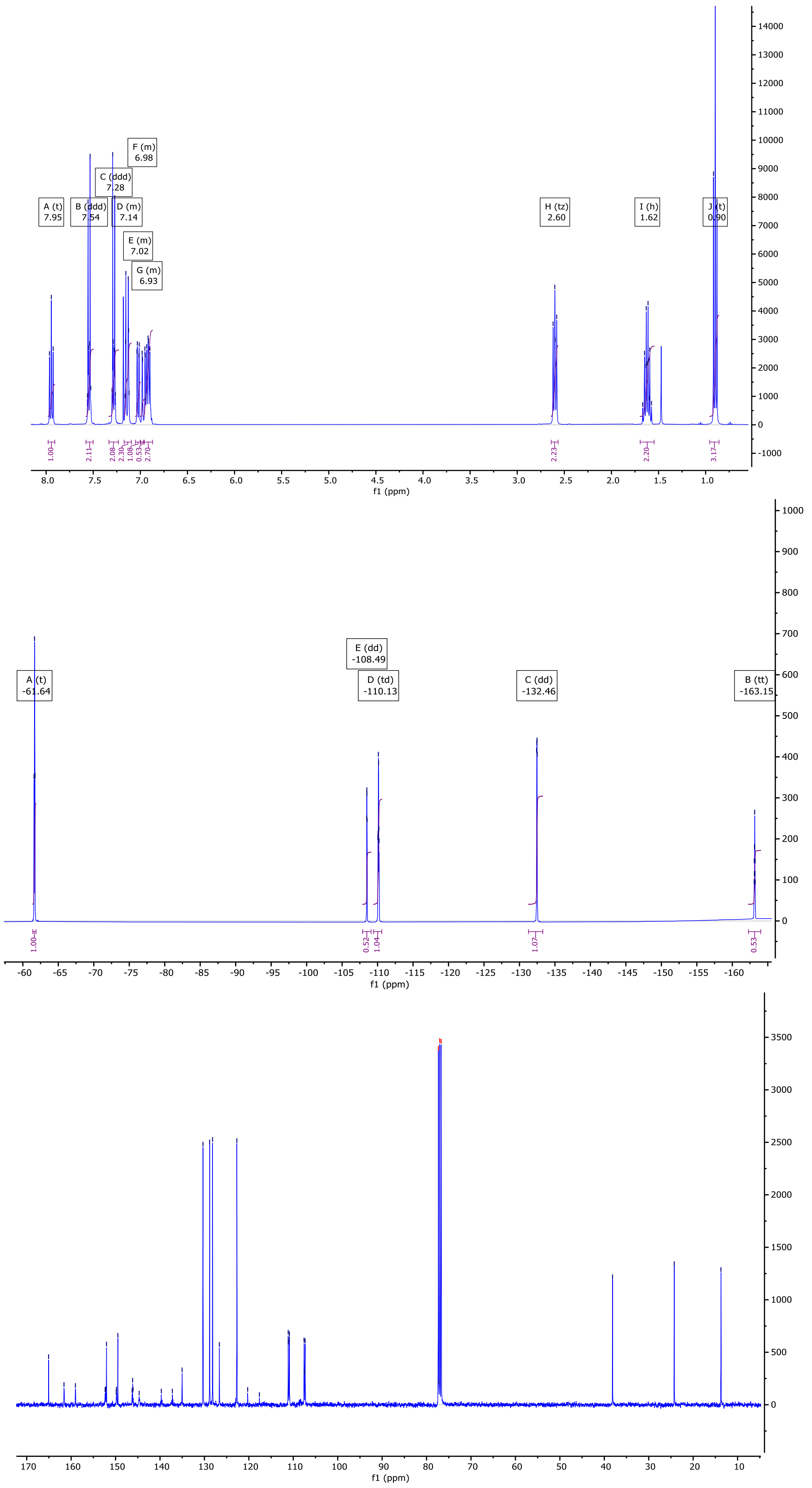

Fig. S27: Example NMR spectra of material **3**: ^{1}H [top], ^{19}F [middle], and $^{13}C\{^{1}H\}$ [bottom].

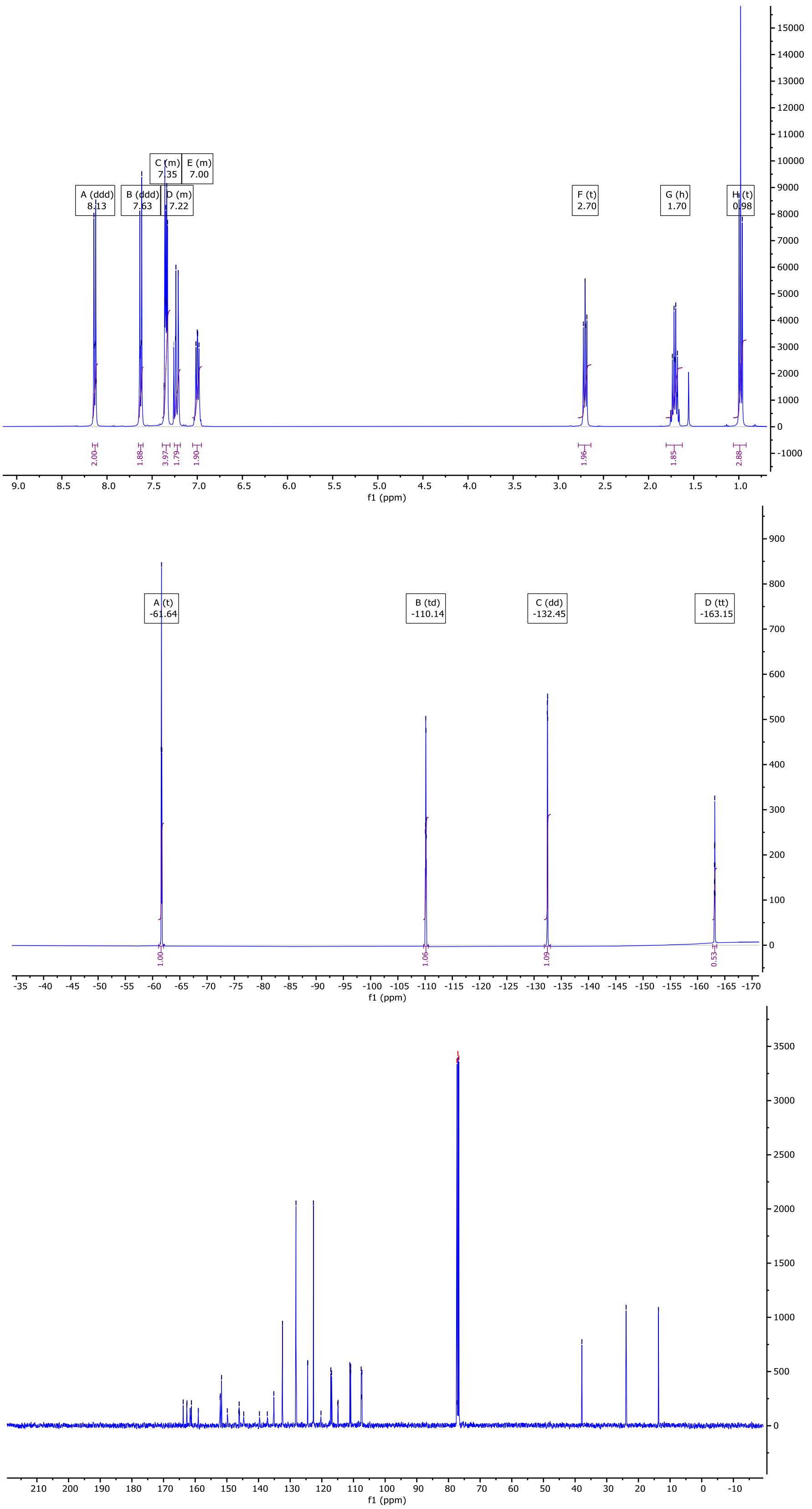

Fig. S28: Example NMR spectra of material **4**: ^{1}H [top], ^{19}F [middle], and $^{13}C\{^{1}H\}$ [bottom].

3. Supplementary References

[1] Martinot-Lagarde Ph., Direct Electrical Measurement of the Permanent Polarization of a Ferroelectric Chiral Smectic C Liquid Crystal, J. Physique Lett. 38, 17 (1977).

[2] K. Miyasato, S. Abe, H. Takezoe, A. Fukuda, and E. Kuze, Direct Method with Triangular Waves for Measuring Spontaneous Polarization in Ferroelectric Liquid Crystals, Jpn J Appl Phys 22, L661 (1983).

[3] X. Chen et al., The Smectic ZA Phase: Antiferroelectric Smectic Order as a Prelude to the Ferroelectric Nematic, PNAS 120, (2023).

[4] Z. Zhang, V. P. Panov, M. Nagaraj, R. J. Mandle, J. W. Goodby, G. R. Luckhurst, J. C. Jones, and H. F. Gleeson, Raman Scattering Studies of Order Parameters in Liquid Crystalline Dimers Exhibiting the Nematic and Twist-Bend Nematic Phases, J Mater Chem C Mater 3, 10007 (2015).

[5] G. Singh, J. Fu, D. M. Agra-Kooijman, J.-K. Song, M. R. Vengatesan, M. Srinivasarao, M. R. Fisch, and S. Kumar, X-Ray and Raman Scattering Study of Orientational Order in Nematic and Heliconical Nematic Liquid Crystals, Phys Rev E 94, 60701 (2016).

[6] C. Meyer, G. R. Luckhurst, and I. Dozov, The Temperature Dependence of the Heliconical Tilt Angle in the Twist-Bend Nematic Phase of the Odd Dimer CB7CB, J Mater Chem C Mater 3, 318 (2015).

[7] M. J. Frisch, G. W. Trucks, H. B. Schlegel, G .E. Scuseria, M. A. Robb, J. R. Cheeseman, G. Scalmani, V. Barone, B. Mennucci, G. A. Petersson, H. Nakatsuji, M. Caricato, X. Li, H. P. Hratchian, A. F. Izmaylov, J. Bloino, G. Zheng, J. L. Sonnenberg, M. Hada, M. Ehara, K. Toyota, R. Fukuda, J. Hasegawa, M. Ishida, T. Nakajima, Y. Honda, O. Kitao, H. Nakai, T. Vreven, J. A. Montgomery Jr., J. E. Peralta, F. Ogliaro, M. J. Bearpark, J. Heyd, E. N. Brothers, K. N. Kudin, V. N. Staroverov, R. Kobayashi, J. Normand, K. Raghavachari, A. P. Rendell, J. C. Burant, S. S. Iyengar, J. Tomasi, M. Cossi, N. Rega, N. J. Millam, M. Klene, J. E. Knox, J. B. Cross, V. Bakken, C. Adamo, J. Jaramillo, R. Gomperts, R. E. Stratmann, O. Yazyev, A. J. Austin,R. Cammi, C. Pomelli, J. W. Ochterski, R. L. Martin, K. Morokuma, V. G. Zakrzewski, G. A. Voth, P. Salvador, J. J. Dannenberg, S. Dapprich, A. D. Daniels, O¨. Farkas, J. B. Foresman ,J. V. Ortiz, J. Cioslowskiand, D. J. Fox, Gaussian 016, Revision E.01, Gaussian, Inc., Wallingford CT, 2016

[8] S. Wang, J. Witek, G. A. Landrum, and S. Riniker, Improving Conformer Generation for Small Rings and Macrocycles Based on Distance Geometry and Experimental Torsional-Angle Preferences, J Chem Inf Model 60, 2044 (2020).

[9] J. Wang, R. M. Wolf, J. W. Caldwell, P. A. Kollman, and D. A. Case, Development and Testing of a General Amber Force Field, J Comput Chem 25, 1157 (2004).

[10] C. I. Bayly, P. Cieplak, W. D. Cornell, and P. A. Kollman, A Well-Behaved Electrostatic Potential Based Method Using Charge Restraints for Deriving Atomic Charges: The RESP Model, J. Phys. Chem 97, 10269 (1993).

[11] C. Lee, eitao Yang, and R. G. Parr, Development of the Colic-Salvetti Correlation-Energy Formula into a Functional of the Electron Density, Phys Rev B 37, 15 (1988).

[12] A. D. Becke, Density-Functional Thermochemistry. III. The Role of Exact Exchange, Journal of Chemical Physics 98, 5648 (1993).

[13] J. Wang, W. Wang, P. A. Kollman, and D. A. Case, Automatic Atom Type and Bond Type Perception in Molecular Mechanical Calculations, J Mol Graph Model 25, 247 (2006).

[14] D. A. Case, T. E. Cheatham, T. Darden, H. Gohlke, R. Luo, K. M. Merz, A. Onufriev, C. Simmerling, B. Wang, and R. J. Woods, The Amber Biomolecular Simulation Programs, J Comput Chem 26, 1668 (2005).

[15] D. Silva and B. F. Vranken, ACPYPE-AnteChamber PYthon Parser InterfacE, Research Notes 5, 367 (2012).

[16] B. Hess, H. Bekker, H. J. C. Berendsen, and J. G. E. M. Fraaije, LINCS: A Linear Constraint Solver for Molecular Simulations, J Comput Chem 18, 1463 (1997).

[17] S. Nosé and M. L. Klein, Constant Pressure Molecular Dynamics for Molecular Systems, Mol Phys 50, 1055 (1983).

[18] M. Parrinello and A. Rahman, Polymorphic Transitions in Single Crystals: A New Molecular Dynamics Method, J Appl Phys 52, 7182 (1981).

[19] W. G. Hoover, Canonical Dynamics: Equilibrium Phase-Space Distributions, Phys Rev A (Coll Park) 31, 1695 (1985).

[20] S. Nosé, A Molecular Dynamics Method for Simulations in the Canonical Ensemble, Mol Phys 52, 255 (1984).

[21] R. T. McGibbon, K. A. Beauchamp, M. P. Harrigan, C. Klein, J. M. Swails, C. X. Hernández, C. R. Schwantes, L. P. Wang, T. J. Lane, and V. S. Pande, MDTraj: A Modern Open Library for the Analysis of Molecular Dynamics Trajectories, Biophys J 109, 1528 (2015).

[22] R. J. Mandle, Implementation of a Cylindrical Distribution Function for the Analysis of Anisotropic Molecular Dynamics Simulations, PLoS One 17, (2022).

[23] N. Sebastián et al., Polarization Patterning in Ferroelectric Nematic Liquids via Flexoelectric Coupling, Nat Commun 14, 3029 (2023).

[24] R. J. Mandle, N. Sebastián, J. Martinez-Perdiguero, and A. Mertelj, On the Molecular Origins of the Ferroelectric Splay Nematic Phase, Nat Commun 12, 4962 (2021).

[25] X. Chen et al., Observation of a Uniaxial Ferroelectric Smectic A Phase, Proceedings of the National Academy of Sciences 119, (2022).

[26] H. Takezoe, E. Gorecka, and M. Čepič, Antiferroelectric Liquid Crystals: Interplay of Simplicity and Complexity, Rev Mod Phys 82, 897 (2010).

[27] J. W. Goodby, C. Tschierske, P. Raynes, H. Gleeson, T. Kato, and P. J. Collings, Handbook of Liquid Crystals, 2nd ed., Vol. 4 (Wiley, 2014).

[28] J. Xiang, Y. Li, Q. Li, D. A. Paterson, J. M. D. Storey, C. T. Imrie, and O. D. Lavrentovich, Electrically Tunable Selective Reflection of Light from Ultraviolet to Visible and Infrared by Heliconical Cholesterics, Advanced Materials 27, 3014 (2015).

[29] N. Hayashi, A. Kocot, M. J. Linehan, A. Fukuda, J. K. Vij, G. Heppke, J. Naciri, S. Kawada, and S. Kondoh, Experimental Demonstration, Using Polarized Raman and Infrared Spectroscopy, That Both Conventional and de Vries Smectic-A Phases May Exist in Smectic Liquid Crystals with a First-Order $A\text{\ensuremath{-}}{C}^{*}$ Transition, Phys Rev E 74, 51706 (2006).

[30] T. Raistrick, Z. Zhang, D. Mistry, J. Mattsson, and H. F. Gleeson, Understanding the Physics of the Auxetic Response in a Liquid Crystal Elastomer, Phys Rev Res 3, 23191 (2021).

[31] A. Sanchez-Castillo, M. A. Osipov, S. Jagiella, Z. H. Nguyen, M. Kašpar, V. Hamplovǎ, J. Maclennan, and F. Giesselmann, Orientational Order Parameters of a de Vries--Type Ferroelectric Liquid Crystal Obtained by Polarized Raman Spectroscopy and x-Ray Diffraction, Phys Rev E 85, 61703 (2012).

[32] N. Hayashi, A. Kocot, M. J. Linehan, A. Fukuda, J. K. Vij, G. Heppke, J. Naciri, S. Kawada, and S. Kondoh, Experimental Demonstration, Using Polarized Raman and Infrared Spectroscopy, That Both Conventional and de Vries Smectic-A Phases May Exist in Smectic Liquid Crystals with a First-Order $A\text{\ensuremath{-}}{C}^{*}$ Transition, Phys Rev E 74, 51706 (2006).

[33] N. Hayashi and T. Kato, Investigations of Orientational Order for an Antiferroelectric Liquid Crystal by Polarized Raman Scattering Measurements, Phys Rev E 63, 21706 (2001).

[34] A. Kocot et al., Observation of the de Vries Behavior in SmA* Phase of a Liquid Crystal Using Polarised Raman Scattering and Infrared Spectroscopy, J Chem Phys 147, 094903 (2017).

[35] C. A. Parton-Barr, H. F. Gleeson, and R. J. Mandle, Room-Temperature Ferroelectric Nematic Liquid Crystal Showing a Large and Diverging Density, Soft Matter 20, 672 (2024).
